# Cryptic diversity in *Ptyodactylus* (Reptilia: Gekkonidae) from the northern Hajar Mountains of Oman and the United Arab Emirates uncovered by an integrative taxonomic approach

**DOI:** 10.1371/journal.pone.0180397

**Published:** 2017-08-02

**Authors:** Marc Simó-Riudalbas, Margarita Metallinou, Philip de Pous, Johannes Els, Sithum Jayasinghe, Erika Péntek-Zakar, Thomas Wilms, Saleh Al-Saadi, Salvador Carranza

**Affiliations:** 1 Institute of Evolutionary Biology (CSIC-Universitat Pompeu Fabra), Passeig Marítim de la Barceloneta 37–49, Barcelona, Spain; 2 Breeding Centre for Endangered Arabian Wildlife, Environment and Protected Areas Authority, Sharjah, UAE; 3 Institute of Animal Science, Biotechnology and Nature Conservation, University of Debrecen, Debrecen, Hungary; 4 Allwetterzoo Münster, Sentruper Str. 315, Münster, Germany; 5 Ministry of Environment and Climate Affairs, Thaqafah Street, Muscat, Oman; State Museum of Natural History, GERMANY

## Abstract

The Hajar Mountains of south-eastern Arabia form an isolated massif surrounded by the sea to the east and by a large desert to the west. As a result of their old geological origin, geographical isolation, complex topography and local climate, these mountains provide an important refuge for endemic and relict species of plants and animals. With 19 species restricted to the Hajar Mountains, reptiles are the vertebrate group with the highest level of endemicity, becoming an excellent model for understanding the patterns and processes that generate and shape diversity in this arid mountain range. The geckos of the *Ptyodactylus hasselquistii* species complex are the largest geckos in Arabia and are found widely distributed across the Arabian Mountains, constituting a very important component of the reptile mountain fauna. Preliminary analyses suggested that their diversity in the Hajar Mountains may be higher than expected and that their systematics should be revised. In order to tackle these questions, we inferred a nearly complete calibrated phylogeny of the genus *Ptyodactylus* to identify the origin of the Hajar Mountains lineages using information from two mitochondrial and four nuclear genes. Genetic variability within the Hajar Mountains was further investigated using 68 specimens of *Ptyodactylus* from 46 localities distributed across the entire mountain range and sequenced for the same genes as above. The molecular phylogenies and morphological analyses as well as niche comparisons indicate the presence of two very old sister cryptic species living in allopatry: one restricted to the extreme northern Hajar Mountains and described as a new species herein; the other distributed across the rest of the Hajar Mountains that can be confidently assigned to the species *P*. *orlovi*. Similar to recent findings in the geckos of the genus *Asaccus*, the results of the present study uncover more hidden diversity in the northern Hajar Mountains and stress once again the importance of this unique mountain range as a hot spot of biodiversity and a priority focal point for reptile conservation in Arabia.

## Introduction

Understanding and quantifying biological diversity is imperative if we want to be able to explain and, ultimately, conserve it. Nevertheless, despite more than 250 years of taxonomic work, we still lack precise estimates of the number of species living on the planet. This uncertainty is typically attributed to inadequate knowledge of small-sized species with reduced distribution ranges concentrated in hotspots and less explored areas of our planet such as the deep sea and soil [1,2]. Recently, molecular methods have revealed that cryptic species, that is, two or more morphologically similar species erroneously classified under the same species’ name, contribute another layer of uncertainty toward realistic estimates of our planet’s diversity [3,4]. One important aspect of crypsis is its potential impact on conservation planning: species that are considered common, widely distributed and of low conservation priority may actually contain multiple morphologically similar species, each with small ranges and therefore of high conservation concern [5–7]. Given the great need for cataloguing and conserving our biota, uncovering cryptic species should be a high priority research so that many of these evolutionary units are not lost before they can be described [1].

It is generally accepted that the tropical regions, which hold the highest biodiversity, also have the greatest potential for harbouring cryptic species, but given the complexity of these environments and their inaccessibility considerable time and effort will be required for their discovery [4]. At the other extreme are deserts and arid regions, which comprise approximately 18% of the earth’s surface [8] and have relatively lower levels of species richness [9]. However, scattered across these plains are isolated mountain ranges with diverse physical and climatic environments [10]. Generally, these mountains have been poorly explored and given their age and physiographical features they may be important sources of diversification, especially for reptiles, which are successful inhabitants of arid environments [11]. In fact, detailed studies from the Atlas Mountains in North Africa [10,12–15] and the Hajar and south-western Mountains in Arabia [6,7,16–18] seem to bear this out.

**Fig 1:**
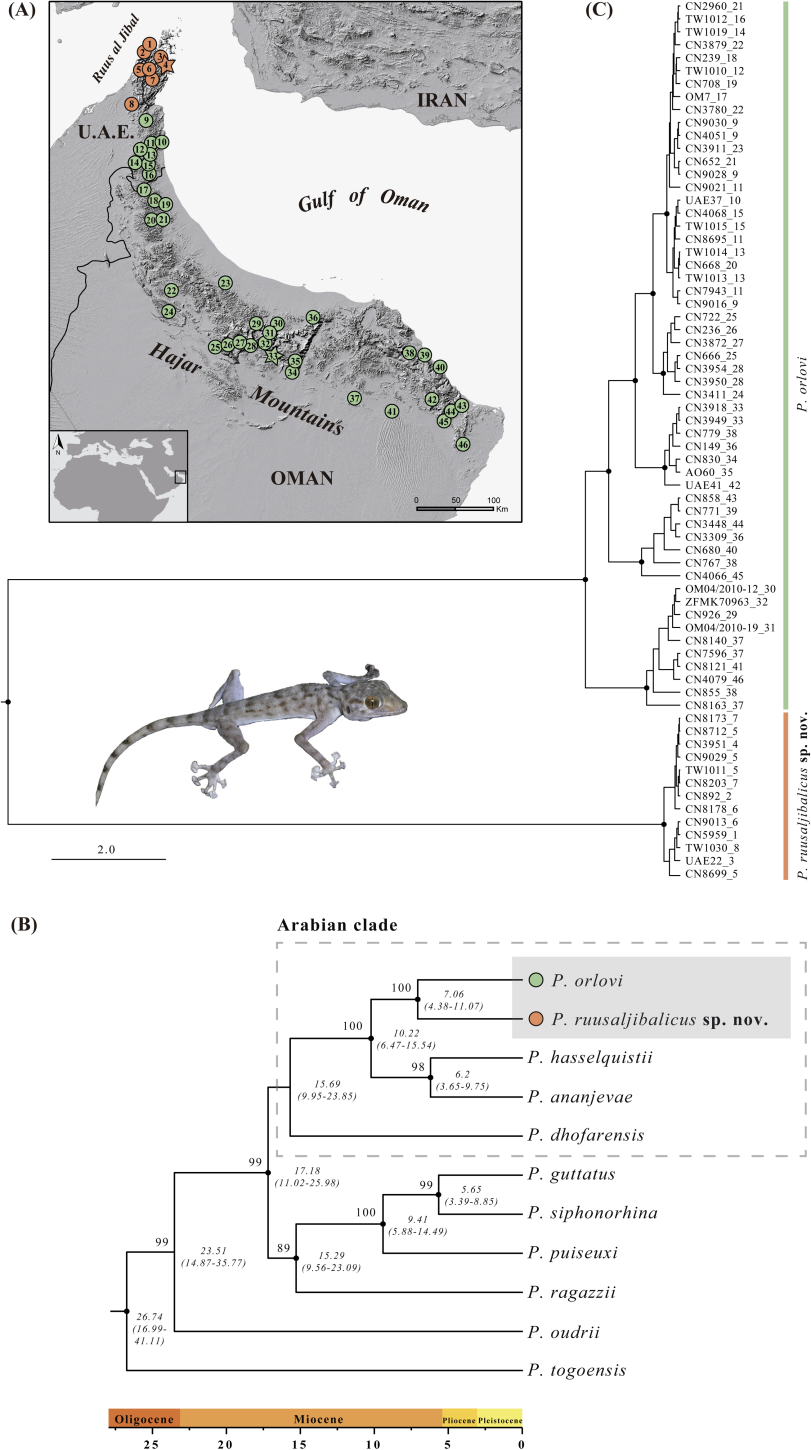
Geographical distribution and phylogenetic relationships of the two species from the Hajar Mountains. (A) Map of the study area showing the localities of examined material. Type localities are marked with star symbols of the same color as the species. Information of all the samples included can be found in S1 Table. Maps were drawn using QGIS v.2.8 (available at http://www.qgis.org; digital elevation model freely available at http://earthobservatory.nasa.gov/). (B) Bayesian inference tree of 11 species of *Ptyodactylus* based on the concatenated sequences of two mitochondrial (*12S* and *cytb*) and four nuclear (*c-mos*, *MC1R*, *ACM4* and *RAG2*) genes. Black dots indicate posterior probability values ≥0.95 in the Bayesian analysis while bootstrap values ≥70% in the maximum likelihood analysis are shown next to the nodes (see S1 Fig). Age estimates are in italics below the nodes and include the mean and the HPD 95% confidence interval in brackets. The tree was rooted using one specimen of *Asaccus gallagheri* (not included in the figure). (C) Bayesian inference tree of 68 *Ptyodactylus* based on the concatenated sequences of two mitochondrial (*12S* and *cytb*) and four nuclear (*c-mos*, *MC1R*, *ACM4* and *RAG2*) genes. Black dots indicate posterior probability values ≥0.95. Each sequence is labelled with the specimen code followed by the locality code (see Fig 1A). Detailed information on the samples included in both phylogenetic trees is given in S1 Table. Inset picture shows a specimen of *P*. *ruusaljibalicus* **sp. nov**.

The Hajar Mountains are the largest mountain range in eastern Arabia, forming a 650 km arc that runs parallel to the coast of the Gulf of Oman (Fig 1A). Most of this region is within Oman but a small section, just south of the Musandam Governorate, is included in the United Arab Emirates. The mountains reach over 2,000 m above sea level in the Ruus al Jibal and over 3,000 m at Jebel Shams, and they are thus high enough to influence local climate significantly. Despite the increased rainfall compared to the surrounding arid lowland regions, average annual precipitation in the Hajar Mountains is still low (less than 300 mm), evapotranspiration is very high (three to four times the annual rainfall) and the barren nature of the terrain classifies it as a mountain desert [19,20]. The geological history of the Hajar Mountains is very complex but at the same time relatively well known. Like other mountain ranges in Arabia, orogeny started in the Oligocene triggered by the opening of the Gulf of Aden, with mountain building extending well into the Miocene, up to 4–6 Ma [21,22]. As a result of its isolation from other mountain ranges in Arabia (the nearest mountains, the Dhofar Mountains of southern Oman, are 700 km away), climatic differences occur between the lowland and the highland areas along with a complex geomorphology. The Hajar mountains are one of the regions with the highest biological diversity of the whole Arabian Peninsula, including numerous endemic plants and animals (e.g. [19] and references therein; [6,7,23–26]). Of all the vertebrates that inhabit the Hajar Mountains, the reptiles are the group that presents the highest level of endemicity, with 19 species found nowhere else in the world [6,7,17,23,26–31].

Within the endemic reptiles of the Hajar Mountains, the geckos of the genus *Ptyodactylus* Goldfuss, 1820 are one of the most conspicuous elements of the reptile fauna, being distributed across the whole mountain range, from sea level up to 2,464 m in elevation ([30]; S. Carranza pers. observ.). They are the largest geckos of the Hajar Mountains, measuring up to 98 mm of snout-vent length, easily recognizable by their fan-like terminal pads (commonly known as Fan-footed geckos). They are found active on cliffs, boulders, rock-bedded wadis (gorges) and caves during the night, although they can also be seen hiding inactive during the day [32,33]. *Ptyodactylus* species are able to produce different types of calls composed of a series of loud clicks that are used for communication [34]. Geographical variation within the Arabian populations of *Ptyodactylus* was first reported by Arnold [28,32]. However, the high level of morphological similarity between the different species of the genus has historically complicated taxonomic work in this group ([35–38], among others). A recent multilocus phylogenetic analysis by Metallinou et al. (2015) [17] shed some light on the systematics of the African taxa and detected unexpectedly high levels of genetic diversity within what they referred to as the *Ptyodactylus hasselquistii* species complex. This is a mainly Arabian species complex composed by *P*. *hasselquistii* sensu stricto (Donndorff, 1789), type locality El Cairo, Egypt; *P*. *ananjevae* Nazarov, Melnikov and Melnikova, 2013, type locality Al Mudawwarah, southern Jordan; *P*. *dhofarensis* Nazarov, Melnikov and Melnikova, 2013, type locality Wadi Ayun, Dhofar, southern Oman; *P*. *orlovi* Nazarov, Melnikov and Melnikova, 2013, type locality Wadi Tanuf, central Hajar Mountains, northern Oman and several undescribed deep phylogenetic lineages distributed across the Arabian Mountains, including one lineage in the Ruus al Jibal, in the extreme northern Hajar Mountains.

In this study, we have sampled specimens of *Ptyodactylus* from localities across the entire Hajar Mountains with the objective of clarifying their systematics and biogeography. The results of the molecular, morphological and ecological analyses indicate the presence of two very old sister cryptic species living in allopatry: one restricted to the northern tip of the Hajar Mountains and described as a new species herein; the other distributed across the rest of the Hajar Mountains that can be confidently assigned to the species *P*. *orlovi*. Similar to recent findings in the geckos of the genus *Asaccus* [7], the results of the present study uncover more hidden diversity in the northern Hajar Mountains and stress once again the importance of this unique mountain range as a hot spot of biodiversity and a priority focal point for reptile conservation in Arabia.

## Materials and methods

### Ethics statement

No in vivo experiments were performed. Specimens were collected and manipulated with the authorization and under strict control, permission of the governments of Oman (Ministry of Environment and Climate Affairs, MECA) and the United Arab Emirates (Environment and Protected Areas Authority, Government of Sharjah) that approved the study. Specimens were captured and processed following the guidelines and protocols stated in the collecting permits and agreements obtained from the competent authorities of Oman and the United Arab Emirates (see references below). Members of the government supervised collecting activities. All efforts were made to minimize animal suffering. All the necessary collecting and export permits for this study in Oman were issued by the Nature Conservation Department of the Ministry of Environment and Climate Affairs, Oman (Refs: 08/2005; 16/2008; 38/2010; 12/2011; 13/2013; 21/2013) and the research in the United Arab Emirates was done under the supervision and permission of the Environment and Protected Areas Authority, Government of Sharjah. This research is not Institutional. As a result of the characteristics of this study and the total control and compliance with the laws, regulations and procedures of this kind of biodiversity studies in Oman and the United Arab Emirates, it did not need the approval by an Institutional Animal Care and Use Committee (IACUC) or ethics committee.

### Molecular analyses

#### Molecular samples, DNA extraction and amplification

A total of 77 individuals of *Ptyodactylus* and one *Asaccus* (*A*. *gallagheri*) were included in the molecular study. A list of all specimens with their taxonomic identifications, sample codes, voucher references, geographical distribution data and GenBank accession numbers for all sequenced genes is presented in S1 Table.

Genomic DNA was extracted from ethanol-preserved tissue samples using the SpeedTools Tissue DNA Extraction kit (Biotools, Madrid, Spain) and six genetic markers were PCR-amplified and sequenced in both directions: the ribosomal 12S rRNA (*12S*) and cytochrome b (*cytb*) mitochondrial gene fragments, and four nuclear fragments of the genes encoding the oocyte maturation factor MOS (*c-mos*), the melanocortin 1 receptor (*MC1R*), the acetylcholine receptor M4 (*ACM4*) and the recombination activating protein 2 (*RAG2*). Primers, PCR conditions and source references for the amplification of all fragments are listed in S2 Table.

#### Sequence analysis

Geneious Pro v.8.0.3 (Biomatters Ltd.) was used for assembling and editing the chromatographs manually. All coding fragments were translated into amino acids and no stop codons were observed. Heterozygous positions for the nuclear coding gene fragments were identified based on the presence of two peaks of approximately equal height at a single nucleotide site in both strands and were coded in both alleles according to IUPAC ambiguity codes. DNA sequences were aligned for each gene independently using the online application of MAFFT v.7 [39] with default parameters (Auto strategy, Gap opening penalty: 1.53, Offset value: 0.0). For the *12S* ribosomal fragment the Q-INS-i strategy was applied, in which information on the secondary structure of the RNA is taken into account. SEQPHASE [40] was used to convert the input files, and the software PHASE v.2.1.1 to resolve phased haplotypes [41]. Default settings of PHASE were used except for phase probabilities that were set to ≥ 0.7 (see [42]). Phased sequences of the nuclear genes were only used for the network analyses. Inter and intra-specific uncorrected *p*-distances with pairwise deletion were estimated for both mitochondrial gene fragments independently using MEGA v.7 [43].

#### Phylogenetic analyses and estimation of divergence times

Two different datasets were used for the phylogenetic analyses. Dataset 1 was assembled to infer the position of the populations from the Hajar Mountains of Oman and the UAE (the object of our study) in a nearly complete calibrated phylogeny of the genus *Ptyodactylus*. This dataset consisted of 11 *Ptyodactylus* specimens (one of: *P*. *togoensis* Tornier 1901, *P*. *ananjevae*, *P*. *dhofarensis*, *P*. *guttatus* Heyden, 1827, *P*. *hasselquistii*, *P*. *oudrii* Lataste, 1880, *P*. *puiseuxi* Boutan, 1893, *P*. *ragazii* Anderson, 1898, and *P*. *siphonorhina* Anderson, 1896 and two of *Ptyodactylus* from the Hajar Mountains of Oman and the UAE: one *P*. *orlovi* and one specimen of the new species described herein) plus one specimen of *Asaccus gallagheri* that was used as outgroup based on published evidence [44–48]. Dataset 1 included all described species of *Ptyodactylus*, with the only exception of *P*. *homolepis* Blanford, 1876 from south Pakistan [17,49], only known from the types and for which DNA sequences are not available. All *Ptyodactylus* specimens from dataset 1 were carefully chosen, so that they were either from the type locality of each included species or very near to it (S1 Table; see also [17]). Dataset 2 was assembled with the aim of studying in detail the phylogenetic relationships and the biogeographic patterns of *Ptyodactylus* from the Hajar Mountains of Oman and the UAE. This dataset included 68 specimens collected from 46 localities distributed across the Hajar Mountains (Fig 1A and S1 Table).

Dataset 1 was analysed with maximum likelihood (ML) and Bayesian inference (BI) methods, whereas dataset 2 was only analysed with Bayesian inference, including only ingroup sequences (all samples from the Hajar Mountains of Oman and the UAE). The best-fit partitioning scheme and models of molecular evolution for datasets 1 and 2 were selected with PartitionFinder v.1.1.1 [50] with the following settings: branch lengths linked, only models available in BEAST evaluated, initial partitions by gene, BIC model selection criterion applied and all partition schemes analysed. The partition scheme and models of sequence evolution selected were *12S*+*cytb*, GTR+I+G; *c-mos*+*ACM4*+*RAG2*, HKY+G and *MC1R*, HKY+I for dataset 1 and 1*2S*+*cytb*, HKY+G; *c-mos*+*ACM4*+*RAG2*, HKY+I and *MC1R*, TN+I+G dataset 2. ML analyses of dataset 1 were performed in RAxML v.7.4.2 [51] as implemented in raxmlGUI [52] with 100 random addition replicates, using the GTR+G model of sequence evolution and independent model parameters for each gene partition. Reliability of the ML tree was assessed by bootstrap analysis [53] including 1,000 replications.

The software BEAST v.1.7.5 [54] was used for BI phylogenetic inference and dating analyses. Two individual runs of 1x10^8^ generations were carried out for datasets 1 and 2, sampling at intervals of 10,000 generations. Models and prior specifications applied were as follows (otherwise by default): model of sequence evolution for each partition as selected by PartitionFinder (see above); Speciation Yule (dataset 1), Coalescent Constant Size (dataset 2) process tree prior for the phylogenetic reconstruction; uncorrelated lognormal clock for mitochondrial genes and strict clock for nuclear ones; random starting tree; base substitution prior Uniform (0,100); alpha prior Uniform (0,10). Partitions and clock models were unlinked and the xml file was manually modified to set Ambiguities = “true” for the nuclear gene partitions in order to account for variability in the heterozygous positions, instead of treating them as missing data. Posterior trace plots and effective sample sizes (ESS) of the runs were monitored in Tracer v1.5 [54] to ensure convergence. The results of the individual runs were combined in LogCombiner discarding 10% of the samples and the ultrametric tree was produced with TreeAnnotator (both provided with the BEAST package). An additional run was performed for all the analyses, sampling only from the prior in order to ensure that the data were sufficiently informative.

The lack of internal calibration points in *Ptyodactylus* precluded any direct estimation of the time of the cladogenetic events in our phylogeny. Alternatively, the absolute divergence times were estimated in the BEAST analysis of dataset 1 (see above) applying previously calculated mean rates of molecular evolution for exactly the same two mitochondrial fragments used in this study *12S* (mean: 0.00755, stdev: 0.00247) and *cytb* (mean: 0.0228, stdev: 0.00806) [6]. Despite the problems associated with using evolutionary rates from other organisms for time tree calibration, the rates inferred by Carranza & Arnold, (2012) [6] and applied here correspond with the rates obtained in other independent studies that used different calibration points and different taxa [55,56]. Indeed, the rates by Carranza & Arnold, (2012) [6] have been applied to several different studies for which reliable internal calibration points based on biogeographic events or fossil evidence do not exist [17,26,31,57–62]. Tree nodes were considered strongly supported if they received ML bootstrap values ≥ 70% and posterior probability (pp) support values ≥ 0.95 [63,64].

#### Intraspecific diversity and nuclear allele networks

The pattern of isolation by distance (IBD) within the two species of *Ptyodactylus* from the Hajar Mountains was tested by a correlation between a matrix of pairwise genetic distances of mitochondrial haplotypes (calculated as the uncorrected *p*-distances) and a matrix of the Euclidean geographic distances between localities using a Mantel test with 10,000 permutations as implemented in the Isolation by Distance Web Service v.3.23 [65]. In all these analyses, the frequency and spatial distribution of mitochondrial haplotypes was obtained by collapsing localities within a maximum range of one kilometre.

Statistical parsimony networks on the four nuclear genes were built with the program TCS v.1.21 [66] using default settings (connection limit of 95%). For comparative purposes, only specimens with complete sequence information for all four nuclear genes were included (S1 Table).

### Morphological analyses

#### Morphological samples and variables

A total of 41 alcohol-preserved specimens of *Ptyodactylus* from the Hajar Mountains of Oman and the UAE were examined and included in the morphological analyses. A list of all studied specimens with sex, metric and meristic variables and their MorphoBank accession numbers is presented in S3 Table. All voucher specimens were obtained from the Natural History Museum, London, UK (NHMUK), the Oman Natural History Museum, Muscat, Oman (ONHM) and S. Carranza’s field series housed at the Institute of Evolutionary Biology (IBE), Barcelona, Spain. The following measurements were taken twice on the right side of each specimen by the same person (MSR) using a digital calliper with accuracy to the nearest 0.01 mm and were expressed in millimetres: snout-vent length (SVL), distance from the tip of the snout to the cloaca; axilla-groin length (AGL), distance between the fore and hind limb insertion points; head length (HL), measured ventrally as the distance from the tip of the snout to the retroarticular process of the jaw; head width (HW), measured as the widest point of the head in dorsal view, usually at the level of the temporal region; head depth (HD), measured laterally as the distance from the ventral to the dorsal surface of the head at mid-eye level; eye to nostril distance (END), measured as the distance from the external nares to the anterior margin of the eye; interorbital distance (IOD), narrowest distance between the eyes; orbital diameter (OD), maximal longitudinal length of the eyeball; ear to eye distance (EED), measured as the distance from the posterior margin of the eye to the anterior margin of the ear opening; brachium length (BL), measured from the elbow to the insertion of the forelimb on the anterior part of body; digit IV of the manus (4^th^ finger) (IVM), measured from the base to the tip of the finger; antebrachium length (AL), measured from the wrist to the elbow; thigh length (ThL), measured from knee to the insertion of the hind limb on the posterior side of the body; crus length (CL), measured from the ankle to the knee; digit IV of the pes (4^th^ toe) (IVP), measured from the base to the tip of the finger and tail length (TL), measured only in the holotype and paratypes of the new species because many individuals had an unequal regenerated tail or had lost it. In addition to these morphometric variables, the following pholidotic (meristic) variables were collected using a dissecting microscope: number of rows of enlarged tubercles on the dorsum (TubL); number of cloacal tubercles (TubA), number of supralabial scales (SL); number of infralabial scales (IL); number of lamellas under the 4th finger (Fan4A); number of lamellas under the 4th toe (Fan4P); number of subdigital scales on the 4th finger (LF4); number of subdigital scales on the 4th toe. Juveniles (SVL<65mm) were only used for the pholidosis. The morphological traits where photographed using a Nikon 300 camera with a 60 mm macro-lens, in order to make all the data easily available to the scientific community. The complete collection of 269 high-resolution photographs has been deposited in MorphoBank (Project 1261; http://www.morphobank.org).

#### Univariate and multivariate analyses

Statistical analyses were used to investigate if there were differences in size and shape between *P*. *orlovi* and the new species of *Ptyodactylus* described herein. The 15 morphometric and the eight meristic variables were analysed independently and juvenile specimens were not included in the multivariate analyses of the continuous variables (Tables 1 and 2). After removing eight juveniles, the dataset of continuous variables included 33 specimens, 29 of which (16 males and 13 females) corresponded to *P*. *orlovi*, and only four (three males and one female) to the new species described herein (S3 Table). All variables were log-transformed to increase the homogeneity of variances. As linear body measurements are generally correlated with body size, all 14 morphometric variables (see above) were regressed against SVL in order to extract the body-size effect using the corresponding residues as a shape proxy. A principal component analysis (PCA) was then performed on the correlation matrix of the residuals to visualize the shape variation between both species in a reduced dimensional space. In order to detect which traits contributed to separate the two species in the morphospace, a one-way ANOVA on each principal component was performed. Regarding body size, differences between species were tested using a one-way ANOVA on the log-transformed values of SVL. In addition, pholidotic differences between both species were tested using a one-way ANOVA for each variable for taxonomic purposes (see taxonomic account).

**Table 1:** Descriptive statistics for all morphometric variables examined for *P*. *ruusaljibalicus* sp. nov. and *P*. *orlovi*. Mean ± Standard Deviation (SD) and range (Min–Max) are given. Abbreviations of characters as explained in the Material and Methods and as in S3 Table.

	*P*. *ruusaljibalicus* sp. nov.		*P*. *orlovi*	
	Males (n = 3)	Females (n = 1)	Males (n = 16)	Females (n = 13)
Variable	Mean±SD (Min–Max)	Mean±SD (Min–Max)	Mean±SD (Min–Max)	Mean±SD (Min–Max)
SVL	85.55±4.97 (80.2–90.01)	85.92	82.15±4.58 (73.66–89.46)	78.86±5.51 (67.83–85.1)
AGL	36.38±2.85 (33.76–39.41)	35.27	34.49±1.97 (31.91–38.53)	34.29±2.56 (29.31–37.96)
HL	23.23±1.51 (21.57–24.54)	23.14	22.76±1.08 (20.53–24.72)	21.57±1.35 (19.1–23.35)
HW	15.34±0.52 (14.83–15.86)	16.36	15.3±0.79 (14.1–16.91)	14.53±0.81 (13.44–15.67)
HH	9.52±0.76 (8.7–10.21)	9.65	9.56±0.56 (8.52–10.7)	9.08±0.72 (7.79–10.22)
END	8.27±0.7 (7.49–8.84)	8.32	7.69±0.55 (6.79–8.78)	7.34±0.49 (6.17–8.1)
IOD	7.98±0.77 (7.42–8.87)	8.14	6.66±0.63 (5.9–8.51)	6.37±0.43 (5.65–7.12)
OD	5.76±0.22 (5.63–6.01)	5.45	5.73±0.31 (5.2–6.33)	5.54±0.33 (4.8–6.01)
EED	6.25±0.52 (5.68–6.7)	7.22	6.72±0.54 (5.6–7.48)	6±0.38 (5.47–6.62)
BL	13.63±0.7 (12.8–14.16)	13.76	13.13±0.73 (11.81–14.14)	12.78±0.81 (11.4–14.09)
AL	16.96±0.14 (16.85–17.11)	16.64	15.79±0.88 (13.58–17.03)	15.19±1.04 (13.71–16.95)
IVM	6.94±0.68 (6.42–7.71)	7.68	7.17±0.59 (6.2–8.05)	6.7±0.63 (5.69–8.23)
ThL	20.61±0.23 (20.38–20.84)	17.92	20.93±1.26 (18.76–23.99)	19.72±1.57 (16.74–21.93)
CL	20.43±1.1 (19.17–21.18)	19.84	19.41±1.13 (17.08–21.26)	18.36±1.41 (16.06–20.45)
IVP	8.88±1.35 (7.67–10.34)	9.84	8.39±0.48 (7.84–9.39)	7.82±0.76 (6.97–9.36)

**Table 2:** Descriptive statistics for all meristic variables examined for *P*. *ruusaljibalicus* sp. nov. and *P*. *orlovi*. Mean ± Standard Deviation (SD) and range (Min–Max) are given. Abbreviations of characters as explained in the Material and Methods and as in S3 Table.

	*P*. *ruusaljibalicus* sp. nov.			*P*. *orlovi*		
	Males (n = 3)	Females (n = 1)	Juveniles (n = 5)	Males (n = 16)	Females (n = 13)	Juveniles (n = 3)
Variable	Mean±SD (Min–Max)	Mean±SD (Min–Max)	Mean±SD (Min–Max)	Mean±SD (Min–Max)	Mean±SD (Min–Max)	Mean±S (Min–Max)
TubW	11	11	9.8±0.84 (9–11)	12.69±0.87 (11–14)	12.38±0.87 (11–14)	12.33±0.58 (12–13)
TubA	4	4	4	3.81±1.33 (1–6)	3.15±1.34 (1–5)	4±2 (2–6)
SL	13±1 (12–14)	13	13.4±0.55 (13–14)	14.19±0.66 (13–15)	13.92±0.76 (12–15)	13.67±1.15 (13–15)
IL	12	13	13	12.88±0.72 (12–14)	13.15±0.8 (12–15)	13±1 (12–14)
Fan4A	20	20	20±1.41 (18–22)	20±1.03 (18–22)	19.69±1.11 (18–22)	20±2 (18–22)
LF4	10.67±0.58 (10–11)	9	9.8±0.84 (9–11)	9.38±0.5 (9–10)	9.62±0.51 (9–10)	9.33±0.58 (9–10)
Fan4P	21.33±1.15 (20–22)	22	20.4±0.89 (20–22)	21±1.26 (18–22)	20.77±1.3 (18–22)	20.67±1.15 (20–22)
LT4	10.33±0.58 (10–11)	10	10.6±0.55 (10–11)	10.19±0.66 (9–11)	10.92±0.64 (10–12)	11.33±0.58 (11–12)

Despite some authors already showed significant sexual dimorphism in some species of *Ptyodactylus* [67], sexual dimorphism across the 15 morphometric variables was tested within *Ptyodactylus* populations from the Hajar Mountains. As a result of the low number of available specimens of the new species described herein, differences on body size and shape between sexes were only tested within *P*. *orlovi*, using a one-way ANOVA for each variable. Summary statistics (mean, maximum, minimum and Standard Error) for each character were calculated for all males, females and juveniles included in the present study (Tables 1 and 2). Data analysis and tests of significance were performed using the statistical software XLSTAT-Pro version 2008.6.8 (Copyright Addinsoft 1995–2008 software).

### Ecological analyses

#### Quantifying niche overlap

A total of 192 distribution records (*P*. *orlovi*, n = 162; the new species described herein, n = 30) were assembled from the authors’ database which, apart from the specimens analysed in the present study, also included personal observations as well as precise observations and bibliographic data from Gardner (2013) [30]. Bioclimatic variables (19) were downloaded from the WorldClim database version 1.4 [68] at a resolution of about 1 km. In order to quantify the degree of ecological differentiation between *P*. *orlovi* and the new species described herein, we employed a multivariate analysis framework proposed by Broennimann et al. (2012) [69] implemented in R (R3.1.2, R Development Core Team 2008). Following this framework, we computed multivariate environmental niche overlaps between the two species from the Hajar Mountains employing the two best performing ordination techniques [69]: (1) Principal Component Analysis (PCA) calibrated on the entire environmental space of the study area (termed PCA-env [69]), and (2) Ecological Niche Factor Analysis (ENFA) [70]. We additionally created 100 datasets with 30 random localities for *P*. *orlovi* to explore if the asymmetric dataset affected similarity significance [71]. The framework by Broennimann et al. (2012) [69] implements a modified niche similarity and niche equivalency tests *sensu* Warren et al. (2008) [71] and calculates niche overlap for pairs of species using Schoener’s *D* [72].

#### Microhabitat analysis

A comparison of the microhabitat use by the two endemic species of *Ptyodactylus* from the Hajar Mountains was carried out with 49 observations of adult specimens of *P*. *orlovi* and of 12 specimens of the new species described herein. Observations took place in spring and autumn 2013. The microhabitat was categorized using information from the substrate and distance above ground, two variables that are considered relevant in geckos [32,73,74]. Substrate observations were summarized into four main categories: 1) cliffs and cave fissures, 2) rocks and boulders, 3) ground and 4) tree branches. On the other hand, height above ground was categorized into three intervals: 1) <0.5 m, 2) 0.5–1.5 m and 3) >1.5 m. These data were analysed with the Fisher exact probability test (N≤120) for a contingency table using the web application VassarStats (www.vassarstats.net).

### Species concept

In this manuscript we have adopted the General Lineage Species Concept [75]. This unified species concept considers species as separately evolving metapopulation lineages and treats this property as the single requisite for delimiting species. Other properties, such as phenetic distinguishability, reciprocal monophyly, and pre- and postzygotic reproductive isolation, are not part of the species concept but serve as important lines of evidence relevant to assess the separation of lineages and therefore to species delimitation [76].

### Nomenclatural acts

The electronic edition of this article conforms to the requirements of the amended International Code of Zoological Nomenclature, and hence the new names contained herein are available under that Code from the electronic edition of this article. This published work and the nomenclatural acts it contains have been registered in ZooBank, the online registration system for the ICZN. The ZooBank LSIDs (Life Science Identifiers) can be resolved and the associated information viewed through any standard web browser by appending the LSID to the prefix “http://zoobank.org/”. The LSID for this publication is: urn:lsid:zoobank.org:pub:7A2CAC5B-86F4-4A5C-A860-ECC399F69F08. The electronic edition of this work was published in a journal with an ISSN, and has been archived and is available from the following digital repositories: PubMed Central, LOCKSS.

## Results

### Molecular analyses

Dataset 1 consisted of a concatenated alignment of 2,706 base pairs (bp) for 12 individuals with 603 variable positions (V) and 327 parsimony informative sites (Pi) including the mitochondrial genes *12S* (397 bp; V = 183; Pi = 109) and *cytb* (393 bp; V = 208; Pi = 150) and the nuclear gene fragments *ACM4* (429 bp; V = 45; Pi = 12), *c-mos* (413 bp; V = 56; Pi = 11), *MC1R* (666 bp; V = 89; Pi = 36) and *RAG2* (408 bp; V = 22; Pi = 9). Dataset 2 consisted of a concatenated alignment of 2,684 bp (V = 187; Pi = 159) including the mitochondrial genes *12S* (375 bp; V = 58; Pi = 47) and *cytb* (393 bp; V = 111; Pi = 96) and the nuclear gene fragments *ACM4* (429 bp; V = 2; Pi = 2), *c-mos* (413 bp; V = 8; Pi = 8), *MC1R* (666 bp; V = 3; Pi = 3) and *RAG 2* (408 bp; V = 5; Pi = 3).

The results of the ML and BI analyses of the genus *Ptyodactylus* (dataset 1) are presented in Fig 1B (see also S1 Fig). The two trees were identical, with most of the nodes well supported. The African species *P*. *togoensis* is the sister taxon to all the other *Ptyodactylus* species included in the analysis. The Maghrebian *P*. *oudrii* is the second species to branch off the tree. The position of *P*. *ragazzii* as sister taxon to the northeast African *P*. *siphonorhina* and the two Levant species *P*. *guttatus* and *P*. *puiseuxi* is well supported by both ML and BI analyses. *Ptyodactylus puiseuxi*, *P*. *siphonorhina* and *P*. *guttatus* form a very well supported clade with the latter two species being phylogenetically more closely related. The members of the *Ptyodactylus hasselquistii* species complex *sensu* Metallinou et al. (2015) [17], a clade to which we will refer herein as to the Arabian clade, branch together in a well-supported group only in the BI analysis. Within the Arabian clade, the south Oman *P*. *dhofarensis* is a sister taxon to a group formed by two well-supported clades; a first clade endemic to the Hajar Mountains of Oman and the UAE that includes two species: *P*. *orlovi* and the new species described herein from Ruus al Jibal, in the extreme northern Hajar Mountains; and a second clade formed by *P*. *ananjevae* and *P*. *hasselquistii* (see Fig 1B). Inferred ages indicate that diversification in *Ptyodactylus* started at least 26.7 Ma (95% HPD = 16.99–41.11). The Arabian clade started diversifying 15.69 Ma (95% HPD = 9.95–23.85) and, within it, divergence between the two species from the Hajar Mountains occurred 7.06 Ma (95% HPD = 4.38–11.07).

The results of the phylogenetic analyses of dataset 2 assembled to study in detail the phylogeographic relationships of *Ptyodactylus orlovi* and the new species described herein are shown in Fig 1C. Despite very good sampling across the Hajar Mountains (Fig 1A), the variability within each of the species is relatively low, especially within the Ruus al Jibal populations. Like in Fig 1B, both main lineages are phylogenetically very well differentiated.

**Fig 2:**
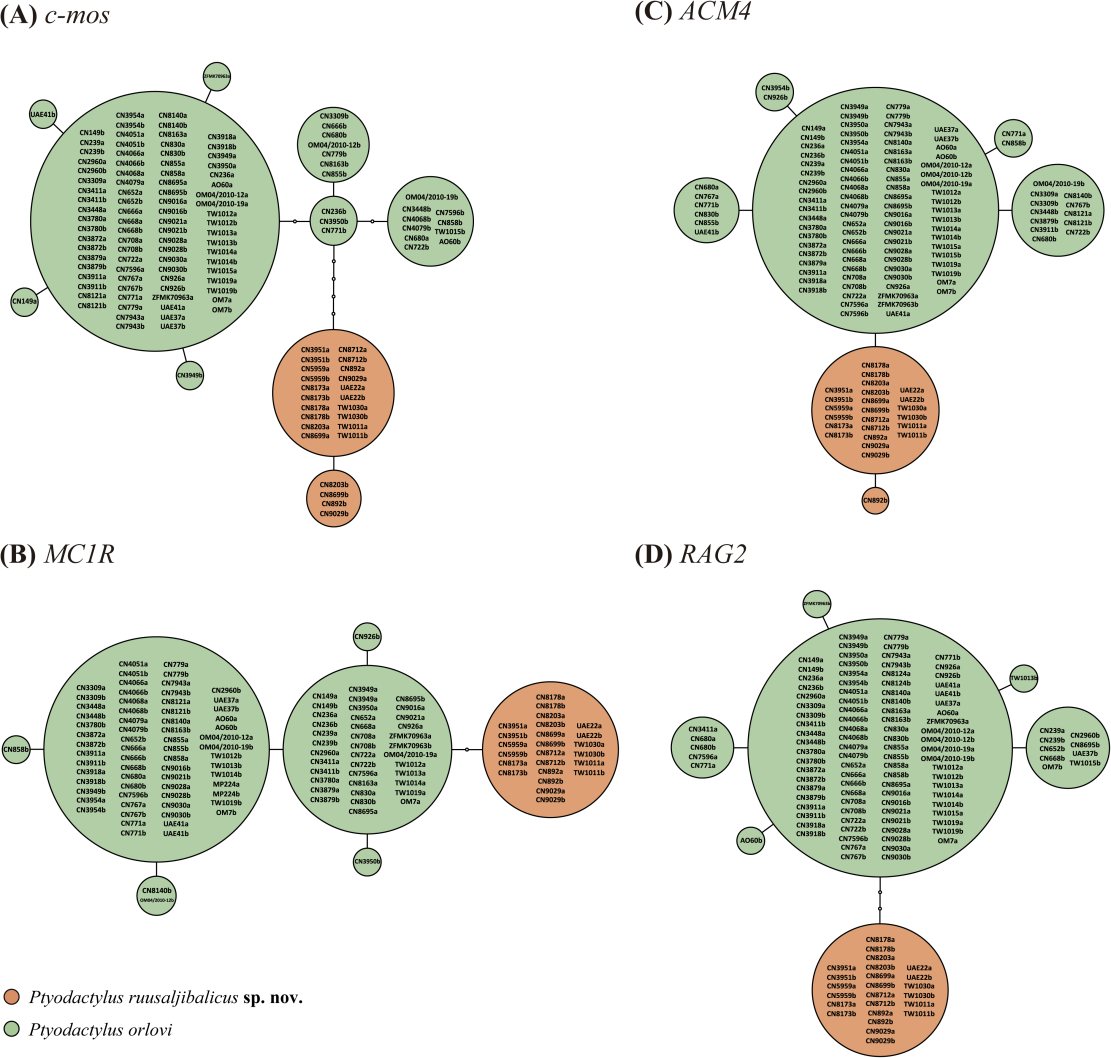
Statistical parsimony nuclear allele networks. (A) *c-mos*, (B) *MC1R*, (C) *ACM4*, (D) *RAG2*. Circle sizes are proportional to the number of individuals and white circles represent mutational steps. Detailed information on the samples included in the networks is given in S1 Table.

The uncorrected genetic distances between the two clades of *Ptyodactylus* from the Hajar Mountains are 10.4±1.5% for the *12S* and 18.8±1.8% for the *cytb*; similar to the genetic divergence between all *Ptyodactylus* species (see Table 3). The level of genetic variability within the Ruus al Jibal populations is 0.2 ±0.1% for the *12S* and 0.8 ±0.2% for the *cytb* and within *P*. *orlovi* is 1.6 ±0.4% for the *12S* and 3.2 ±0.4% for the *cytb*. The results of the haplotype network analyses are presented in Fig 2 and clearly show that, despite the relatively high number of specimens analysed, the clade formed by the populations from the Ruus al Jibal and *P*. *orlovi* do not share a single allele in all four nuclear genes analysed. The populations from Ruus al Jibal include six haplotypes (two in the *c-mos*, one in *MC1R*, two in *ACM4* and one in *RAG2*) and *P*. *orlovi* presents 25 haplotypes (eight in *c-mos*, six in *MC1R*, five in *ACM4* and six in *RAG2*). The different haplotypes of *P*. *orlovi* do not present any geographic structure, being distributed evenly over the various sampling sites (Fig 1A, Fig 2 and S1 Table).

**Table 3:** Uncorrected *p*-distances between all the *Ptyodactylus* species included in this study, using the *12S* (lower left) and *cytb* (upper right) mitochondrial markers.

	1.	2.	3.	4.	5.	6.	7.	8.	9.	10.
**1.** *P*. *ruusaljibalicus* **sp. nov.**		0.188	0.183	0.190	0.239	0.211	0.215	0.236	0.218	0.237
**2.** *P*. *orlovi*	0.104		0.176	0.202	0.227	0.226	0.228	0.244	0.224	0.235
**3.** *P*. *ananjeave*	0.125	0.118		0.152	0.220	0.180	0.181	0.197	0.178	0.205
**4.** *P*. *hasselquistii*	0.133	0.118	0.082		0.248	0.219	0.198	0.200	0.203	0.231
**5.** *P*. *dhofarensis*	0.166	0.162	0.159	0.169		0.207	0.210	0.222	0.201	0.236
**6.** *P*. *ragazzii*	0.180	0.162	0.154	0.175	0.161		0.183	0.212	0.196	0.196
**7.** *P*. *puiseuxi*	0.142	0.132	0.144	0.146	0.143	0.150		0.155	0.166	0.199
**8.** *P*. *siphonorhina*	0.173	0.148	0.154	0.153	0.154	0.164	0.104		0.138	0.225
**9.** *P*. *guttatus*	0.184	0.152	0.153	0.158	0.152	0.152	0.108	0.097		0.202
**10.** *P*. *oudrii*	0.196	0.188	0.176	0.189	0.204	0.187	0.167	0.170	0.183	

The results of the isolation by distance analysis indicate that geographic distance contributes significantly to genetic isolation in *P*. *orlovi* (Mantel’s r = 0.46, *P*<0.001). Even so, many divergent haplotypes co-occur in nearby locations decreasing the correlation between genetic and geographic distances (see S2 Fig). For the new species from the Ruus al Jibal described herein, no pattern of isolation-by-distance was found (Mantel’s r = 0.21, *P* = 0.26) as a result of the incipient genetic variability displayed in a reduced area (about 70 km from north to south and 30 km from east to west) (Fig 1A and S2 Fig). It is important to notice that sensitivity of this test is commonly diminished because the Euclidian distances do not integrate all possible pathways connecting populations considering important orographic barriers like topographic relief of mountains.

### Morphological analyses

**Fig 3:**
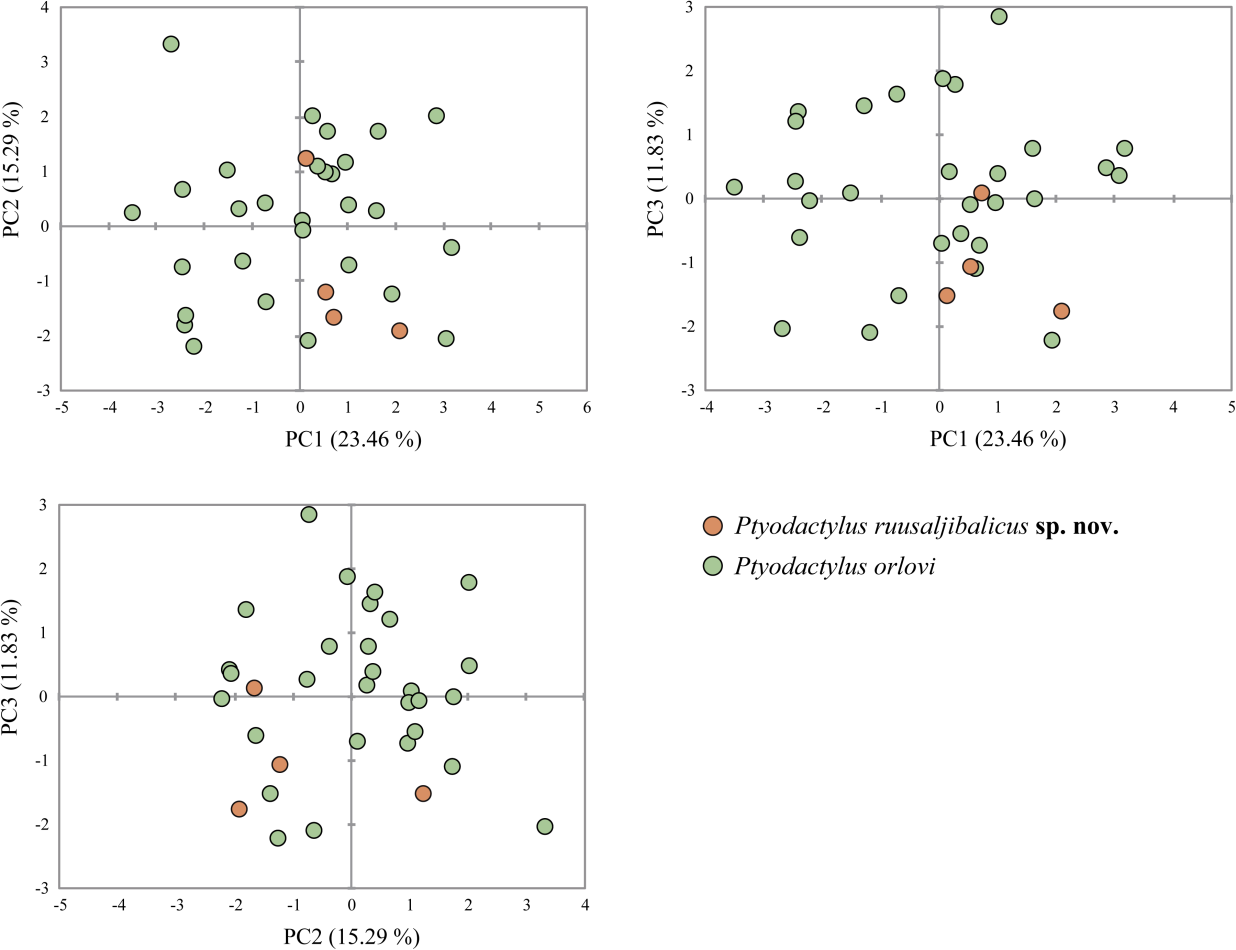
Principal Component Analysis (PCA) of the shape related morphological data. The contribution of the first three components explaining morphological variation is given in brackets. See material and methods for details.

Shape differences between *P*. *orlovi* and the new species described herein are presented in Fig 3 and descriptive statistics for all 23 measured variables are in Tables 1 and 2. Sexual dimorphism on size was not significant (F = 3.085; d.f. = 1; *P* = 0.09). Shape differences between sexes were tested for each character and this test result was only significant for one variable: EED (*P* = 0.001). Consequently, this morphometric character was excluded and both sexes were pooled together in all posterior analyses. Size differences between species were not significant (F = 3.145; d.f. = 1; *P* = 0.086) and shape differentiation tested on the PCA scores of the 13 components was not significant for all of them. Therefore, both species overlap clearly in the morphospace and we cannot differentiate each other with the current morphometric data (Fig 3).

### Ecological analyses

**Fig 4:**
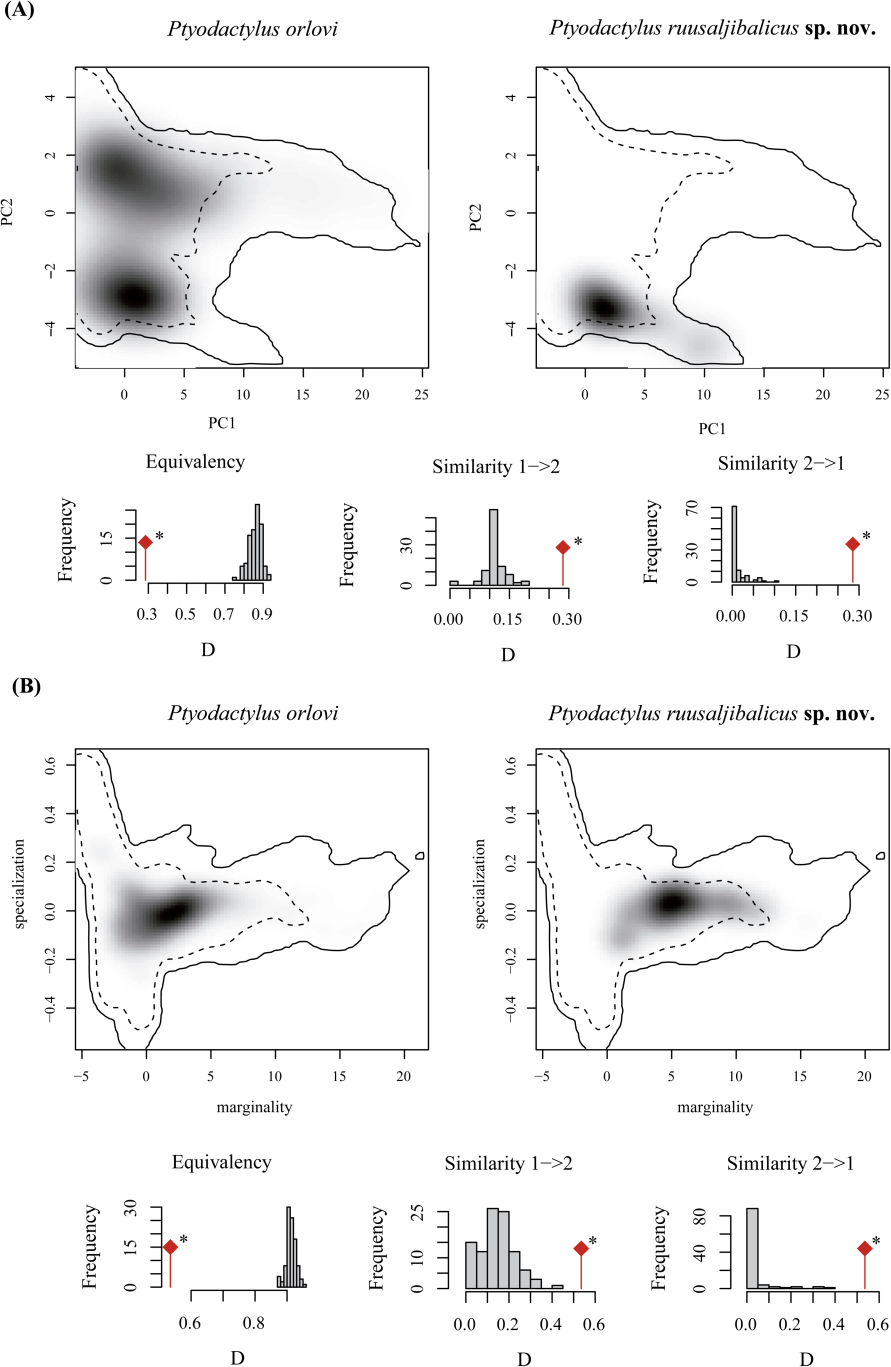
**Visualization of the climatic space occupied by *Ptyodactylus orlovi* (1) and *Ptyodactylus ruusaljibalicus* sp. nov. (2) based on PCA-env (A) and ENFA (B).** (A) The niches of both species are displayed on a multi-dimensional scale represented by the first two axes of a principal component analyses (PCA) summarizing the entire study area. (B) The x-axis shows marginality and the y-axis specialization. In both figures the grey shadings reflect the density of the occurrences of each species by cell. The dashed and solid contour lines illustrate, respectively, 50% and 100% of the available background environment. The significance of the equivalency and similarity tests is shown with an asterisc (*).

The environmental space occupied by *P*. *orlovi* and the new species described herein as determined by PCA-env and ENFA is shown in Fig 4. Niche overlap between the species is low (D = 0.285) for PCA-env to moderate for ENFA (D = 0.535) (following Rödder and Engler 2011 [77]) and the niche equivalency hypothesis was rejected (*P* = 0.02) for both PCA-env and ENFA, indicating that the two species have undergone significant alteration of their environmental niche. The background similarity test, however, shows that the niches of *P*. *orlovi* and the new species are significantly more similar than would be expected given the underlying environmental differences between the regions they inhabit (backgrounds) for both PCA-env and ENFA (*P* = 0.02 in both directions). The randomization test indicates that 13% (new species > *P*. *orlovi*) and 5% (*P*. *orlovi* > new species) of the 100 random replications are non-significant. Overall, the studied macroniches are different but they can predict each other quite well, so there is a degree of niche conservatism.

Field observations did not show significant differences in substrate occupation (*P* = 0.65) between both species. All specimens of the new species described herein were observed inhabiting cliffs and cave fissures (category 1; n = 6) and also rocks and boulders (category 2; n = 6). Similarly, *P*. *orlovi* was mainly observed on cliffs and cave fissures (category 1; n = 28) and rocks and boulders (category 2; n = 19), even though a few specimens where on the ground (category 3; n = 2) and tree branches (category 4; n = 1). Differences in distance above ground were also negligible (*P* = 0.23). *P*. *orlovi* was abundantly found in the three height intervals (<0.5 m; n = 13, 0.5–1.5 m; n = 14 and >1.5 m; n = 23) and, regarding the new species described in this study, only one specimen were found under 0.5 m, 6 specimens between 0.5 and 1.5 m and 5 specimens over 1.5 m.

### Taxonomy

Despite the high level of crypsis between the populations from the Ruus al Jibal and *P*. *orlovi* in the characters studied here, the results of the two mitochondrial and four nuclear gene fragments analysed (Figs 1B, 1C and 2) clearly show that these two lineages have been evolving independently for a long time. As a result of that, and based also on a few morphological traits (see diagnosis below, Tables 1 and 2 and S3 Table), we describe this unnamed population from the extreme northern part of the Hajar Mountain range as a new species.

Family Phyllodactylidae

Genus *Ptyodactylus* Goldfuss, 1820

#### *Ptyodactylus ruusaljibalicus* sp. nov.

**Fig 5:**
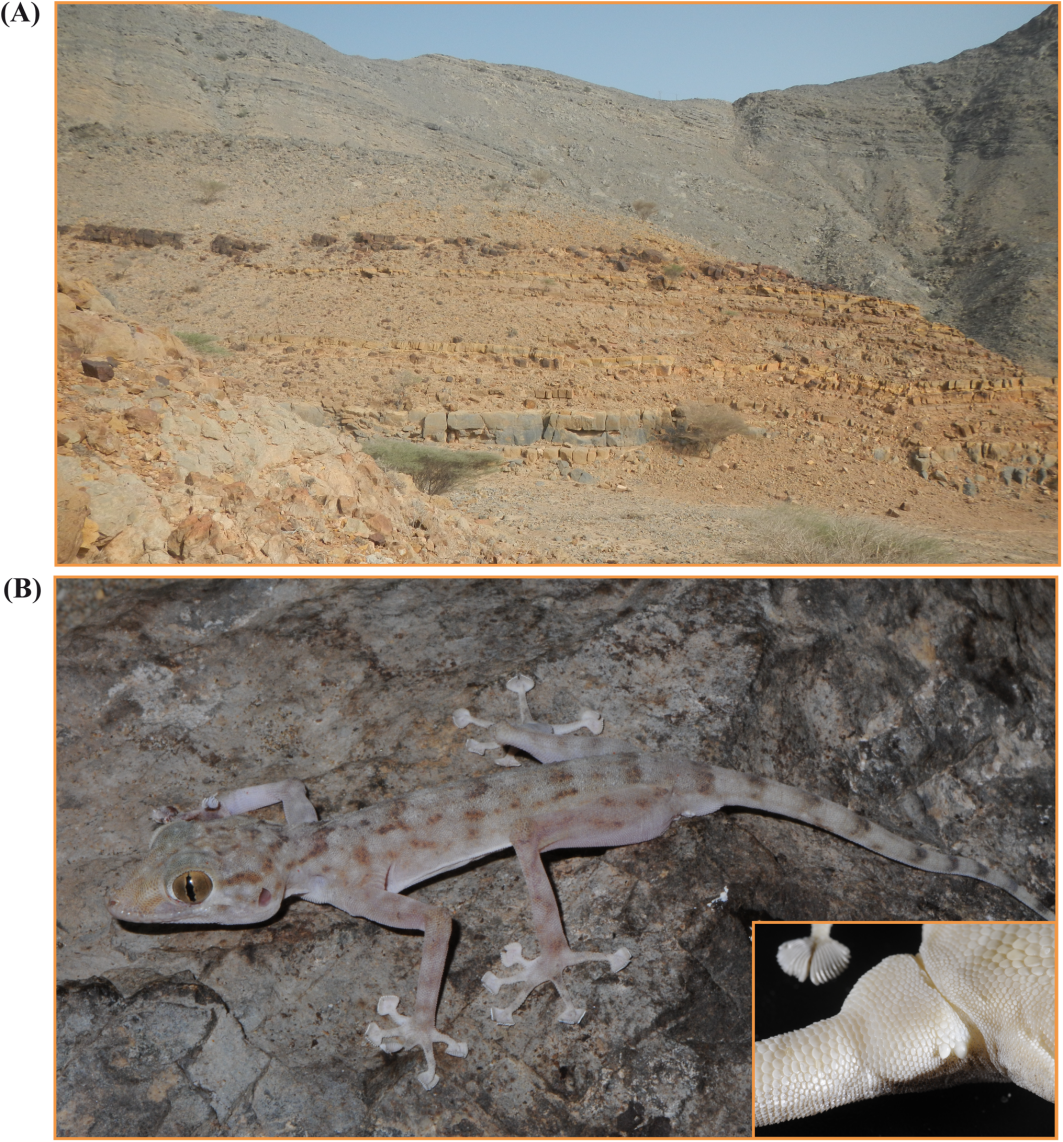
View of the common habitat in the mountainous Ruus al Jibal and general appearance in life of *Ptyodactylus ruusaljibalicus* sp. nov. (A) Rocky habitat in the type locality on the 23^rd^ of April 2013. (B) Holotype of *P*. *ruusaljibalicus* **sp. nov.** (voucher code NHMUK2013.347) including a detail of the cloacal tubercles at the tail base. All photographs taken by Salvador Carranza.

urn:lsid:zoobank.org:act:A2DDA836-4AE9-40F2-9E98-0A224E7A00D0

(Figs 1–5; Tables 1–3; S1 and S2 Figs; S1 and S3 Tables)

MorphoBank M339669–M339739

*Ptyodactylus hasselquistii*. Arnold and Gallagher, 1977: 65 (part.); Arnold, 1977: 106 (part.); Arnold, 1986: 421 (part.); van deer Kooij, 2000: 117 (part.); Sindaco and Jeremcenko, 2008: 123 (part.); Gardner, 2013: 186 (part.).

*Holotype*. NHMUK2013.347, adult male, from Musandam (Oman), 26.04214N 56.36966E WGS84, collected by S. Carranza, M. Metallinou, Ali Alghafri, Sultan Khalifa and Hamed Al Furkani on the 22^nd^ of April 2013 between 12:30–13:30, tissue code CN3951 (MorphoBank M339669–M339684).

*Paratypes*. NHMUK2013.348, adult male, from Musandam (Oman), 26.22711N 56.21312E WGS84, collected by S. Carranza, M. Metallinou, Ali Alghafri, Sultan Khalifa and Hamed Al Furkani on the 21^st^ of April 2013 between 22:10–22:40, tissue code CN5959 (MorphoBank M339685–M339691); IBECN892, adult male from Musandam (Oman), 26.15057N 56.16159E WGS84, collected by S. Carranza, M. Metallinou, Ali Alghafri, Sultan Khalifa and Hamed Al Furkani on the 21^st^ of April 2013 between 23:15–23:45, tissue code CN892 (MorphoBank M339662–M339668); ONHM3743, adult female, from Musandam (Oman), 25.97805N 56.20497E WGS84, collected by S. Carranza, M. Metallinou, J. Smid and R. Vasconcelos on the 24^th^ of October 2013 between 19:42–21:00, tissue code CN8178 (MorphoBank M339698–M339717).

*Other material examined*. Five juveniles listed in S1 and S3 Tables, only considered here for the genetic and ecological analyses and also for the pholidotic traits.

*Etymology*. The specific name “*ruusaljibalicus”* is an adjective that refers to Ruus al Jibal, that means “Heads of the Mountains”, where all the specimens that belong to this species have been found to date and from where the species is probably endemic.

*Diagnosis*. A large size species of the genus *Ptyodactylus* characterized by the following combination of characters: (1) large size with a maximum recorded SVL of 90.01 mm for males and 85.94 mm for females (only one female known); (2) head narrow with elongated snout; (3) 12–13 infralabials and 12–14 supralabials; (4) dorsum with 9–11 irregular longitudinal rows of round, enlarged and slightly keeled tubercles; (5) absence of enlarged tubercles on the dorsal side of the extremities; (6) four prominent cloacal tubercles at the tail base (two on each side); (7) 9–11 subdigital scales on the 4^th^ finger and 10–11 under the 4^th^ toe; (8) 18–22 terminal lamellae under the 4^th^ finger and 20–22 under the 4^th^ toe; (9) in life, uniform light grey dorsum, some specimens with dark brown transverse bands that extended onto the tail. Underside of body and tail ivory-white.

*Ptyodactylus ruusaljibalicus* **sp. nov.** is morphologically very similar to *P*. *orlovi*, its phylogenetic sister taxon (Fig 1B and 1C), and the only species of the genus geographically close to it (Fig 1A). However, the data presented here suggest that *P*. *ruusaljibalicus* **sp. nov.** may be differentiated from *P*. *orlovi* by a lower number of longitudinal rows of enlarged tubercles (9–11 *versus* 11–14; *P*<0.001) that are usually less keeled; cloacal tubercles more prominent, visible dorsally, four in total (two on each side) *versus* 1–6 (less prominent and mostly unevenly distributed); usually lighter dorsal colour and less evident pattern of dark bands and spots on back. On the other hand, it can be clearly differentiated at the genetic level by *p*-distances of 10.4±1.5% in the *12S* and 18.8±1.8% in the *cytb* together with the absence of allele sharing between the two species in the nuclear markers *c-mos*, *RAG2*, *ACM4* and *MC1R* (Fig 2). *Ptyodactylus ruusaljibalicus* **sp. nov.** can be differentiated morphologically from the only species not included in the phylogenetic analyses, *P*. *homolepis*, by its smaller size (max SVL 90.01 mm *versus* 105 mm); by the presence of enlarged and slightly keeled dorsal tubercles on the back (absence of enlarged tubercles in *P*. *homolepis*); and by rostral and first supralabials entering the nostril (nostrils entirely surrounded by swollen nasals which separate them entirely from the rostral and supralabials in *P*. *homolepis*).

*Description of the holotype*. NHMUK2013.347. Data on 15 morphometric and eight meristic variables (see Material and Methods) are provided in S3 Table. Adult male, medium size (SVL 80.2 mm), slender body with relatively elongated extremities. Head relatively narrow with elongated snout (HW/SVL = 0.18, HW = 69% HL). Rostral roughly rectangular; nostrils protuberant defined by rostral, upper labial (both entering broadly in to lower nostril border), and three supranasals in contact with upper border; inner supranasals in contact with rostral and separated from each other by one polygonal scale; 12/14 (right/left) upper labial scales and 12/13 lower labials (MorphoBank M339671). Size and shape of head scales small granular, uniform and non-imbricate with some enlarged tubercles on the back of head and neck. Mental scale elongated separating the first postmental, two postmentals on the right side and three on the left; ear opening vertical, elliptical and elongated; large eyes (ED/SVL = 0.07) with vertical pupil; 11 rows of elongated tubercles on the back, scales slightly keeled; scales on dorsum of body and extremities small, granular, uniform and non-imbricate; scales underside the head and body increasing in size towards the pelvic area, small, granular in the gular region, more elongated and distinctly imbricated on the underside of neck, slightly imbricated between the axilla and groin and larger, rhomboid and imbricated in the pelvic area. Hemipenial bulges very obvious with two cloacal tubercles on each side. Elongated digits with 11 subdigital scales and twenty terminal lamellae under the 4^th^ finger and toe (MorphoBank M339672 and M339674). Non-regenerated tail of similar length to SVL (TL = 80.5 mm; TL/SVL = 1). Tongue partially removed for genetic analyses.

Colouration in alcohol whitish-yellow underneath and pale grey above, with irregular transversal dark bands and spots across the body. Tail with ten dark transversal bands being narrower and increasing in intensity distally; ventral surface of tail pale with no bands extending on to it (Morphobank M339669–M339670). Two thin interrupted dark stripes, one from the back of eye, over the ear, across the neck and on to occipital area, the other starting in the posterior side of ear to shoulders. Colour in life much richer than in the fixed specimen; light grey-ochre with the above-described pattern of marks more evident. Iris in life colourful, golden with dark venations (MorphoBank M339675–M339684).

*Variation*. Data on 15 morphometric and eight meristic variables (see Material and Methods) for all three paratypes, NHMUK2013.348, IBECN892 and ONHM3743 are provided in S3 Table. All the specimens are very similar to each other varying slightly in size related measurements, number of supralabials, terminal lamellae under the 4^th^ toe and number of subdigital scales under the 4^th^ finger and the 4^th^ toe (S3 Table). Tails of paratypes ONHM3743 and NHMUK2013.348 broken at the base, in the latter preserved intact together with the specimen (TL = 85.67). Right side of the head damaged in paratype ONHM3743 (MorphoBank M339705). Tail of IBECN892 regenerated. Main colouration very similar to the holotype, with paratypes IBECN892 and NHMUK2013.348 being much lighter, lacking dorsal and head dark markings (MorphoBank M339662 and M339685 respectively) and paratype ONHM3743 with very weak longitudinal dark bands from neck to pelvic area (MorphoBank M339698–M339701, M339703).

*Distribution and ecology*. Despite intensive sampling across the Hajar Mountain range and other areas in Arabia carried out between 2004 and 2014, *Ptyodactylus ruusaljibalicus* **sp. nov.** has only been found in the Ruus al Jibal region, from the Musandam Peninsula to the Dibba region in the UAE. It can be therefore considered endemic to this distinctive geographical area (Fig 1A). The northernmost and southernmost localities lie approximately 26 km northwest and 58 km south-west of the type locality, respectively. The minimum distance between *Ptyodactylus ruusaljibalicus* **sp. nov.** and *P*. *orlovi* is 23 km by air. *Ptyodactylus ruusaljibalicus* **sp. nov.** inhabits cliffs and cave fissures, rocks and boulders at different heights. The species is mainly nocturnal, although some specimens were out in the shade during the day.

*Conservation status*. Not evaluated.

*Proposal of common names*.

English: Ruus al Jibal fan-footed gecko

Arabic: الأقدام مروحية الجبال رؤوس وزغة

## Discussion

The results of the integration of molecular, morphological and ecological data reveal a very old speciation event within the genus *Ptyodactylus* and highlight another case of endemicity in the northern tip of the Hajar Mountain range [7]. Our discovery is relevant because it shifts attention from the much better explored and studied Jebel Akhdar, in the central section of the Hajar Mountains, a recognized hotspot of biodiversity [78,79] and indicates that other less investigated areas of the Hajar Mountains may be reservoirs of high levels of diversity, especially of cryptic species. With the description of *Ptyodactylus ruusaljibalicus* **sp. nov.** and the presence of *Asaccus margaritae*, *A*. *gardneri* and *A*. *caudivolvulus* [7], this short and narrow mountain stretch of approximately 140 km from north to south and 40 km from east to west (4,350 km^2^) contains four endemic species of reptiles, exactly the same number of endemics inhabiting the Jebel Akhdar in the central Hajar Mountains (*Hemidactylus luqueorum*, *Asaccus platyrhynchus*, *A*. *montanus* and *Pristurus gallagheri*; [30]). The recent descriptions of *Asaccus* species [7] and the present work are very good examples of the potential impact that the lack of taxonomic knowledge (the Linnean shortfall; [80]) can have on conservation planning. Species that are considered common and widely distributed may actually contain multiple species, each with small ranges and of potentially high conservation concern, such as *A*. *caudivolvulus*, the reptile with the smallest distribution range of all 19 reptile species endemic to the Hajar Mountains [5–7].

Despite the broad distribution range of *Ptyodactylus* geckos and the high level of genetic diversity including several described and undescribed species, all its members present very conserved morphologies in terms of body size and especially body shape [17,32,33,73] and overall allopatric distributions, with very few areas where a maximum of two species coexist in sympatry [17,33,38,67,81,82]. A similar case to the geckos of the genus *Hemidactylus* from southwestern Arabia [83] and the *Pristurus rupestris* species complex from the Hajar Mountains [84] and in contrast to other Arabian geckos like *Stenodactylus* [32,55], which show high levels of divergence in body size and shape throughout many areas with three or more species living in sympatry. This morphological stasis in *Ptyodactylus* may be explained by morphological constraints imposed by the use of a similar structural habitat (open rocky surfaces such as cliffs, large boulders and the walls and ceilings of caves; [32]) by all its members. Like all the species in the genus, the two *Ptyodactylus* from the Hajar Mountains are morphologically very similar, up to the point that we are unable to detect any differences in body size and body shape despite an inferred divergence of more than 7 Ma (95% HPD = 4.5–11), representing a clear case of cryptic sister species (sibling species; see [3]). Despite the morphological stasis, the lack of shared haplotypes in the four nuclear genes suggests that there is no signal of gene flow between both species (Fig 2). Furthermore, the great genetic divergence observed in two mitochondrial gene fragments is equivalent to interspecific distances between the other species of the genus (Table 3). This is even more relevant if one takes into account that the two species are geographically very close, the nearest localities being just 23 km from each other (Fig 1A).

Considering the intensive sampling effort across Oman and the UAE between 2004 and 2016 and the large number of samples included in this study from different localities, we can confidently deduce an allopatric distribution pattern like the one shown in Fig 1A, with the new species restricted to the Ruus al Jibal, in the extreme northern Hajar Mountains and *P*. *orlovi* populations widely distributed along the rest of the mountain range. The Ruus al Jibal is often considered a part of the Hajar Mountain arch, but has its own distinctive geology, structure and geomorphology. This relatively small area rises to a maximum elevation of 2,087 m in Jebel al Harim in the north and is separated from the ophiolites of the Oman Mountains, to the south, by the NE-SW Dibba Zone [85] and from the sedimentary sequence of the UAE, to the west by the Musandam-Zagros hinge, sometimes called the “Oman Line”, both zones being zones of deep basement faulting [20]. Although the current geographic ranges of *P*. *ruusaljibalicus* **sp. nov**. and *P*. *orlovi* points towards an association of the isolation of their nearby locations in both sides of the Dibba Zone, it is not possible to present a firm hypothesis with the available data given the complex and dynamic geological history of the area [20,86–90]. Regarding habitat occupation, the two species do not differ in their microniche but show low to moderate niche overlap and non-equivalent macroniches. Our data support Warren et al. (2008) [71] and show that similarity test significance can arise as result of asymmetric datasets (*P*. *orlovi*, n = 162 and *P*. *ruusaljibalicus*, n = 30). However, the similarity tests indicate that the two different macroniches can predict each other quite well, indicating some degree of niche conservatism.

It has been suggested that in the early stages of speciation, species tend to diversify into distinct (mostly allopatric) macroniches while maintaining a similar microniche (e.g. presenting extensive overlap in their body size and shape) [91]. Indeed, low levels of morphological change may be a common pattern between strictly allopatric species in which resource partitioning between them is not needed [92]. Because there are no significant differences in their body size and shape, we assume that stabilising selection on adaptive traits related to exploit a particular structural niche might be maintaining an external conserved morphology [17,93,94]. Moreover, it has been shown that evolution under extreme environmental conditions can limit morphological change, as in the case of cryptic species found in Arctic tundra, underwater karsts and deep-sea environments [3]. On the other hand, many of these cryptic species can differentiate each other by non-visual signals as a result of their morphological stasis [3]. Organisms that use non-visual signals for communication (e.g. sound, vibration, pheromones or electrical signals) are most likely to harbour cryptic species because shifts in signals conveyed in these modalities do not usually involve morphological change [95]. Since femoral or preanal pores for chemical communication are not present in *Ptyodactylus* geckos, future studies should be directed to test the role of other non-visual signals such as vocal sounds in speciation [33,96,97].

## Supporting information

S1 Fig ML tree of 11 *Ptyodactylus* species. The phylogeny is based on the concatenated sequences of two mitochondrial (*12S* and *cytb*) and four nuclear (*c-mos*, *MC1R*, *ACM4* and *RAG2*) gene fragments. Bootstrap values ≥70% of the ML analysis are shown next to the nodes.
(TIF)

S2 Fig IBD analyses for *Ptyodactylus orlovi* and *Ptyodactylus ruusaljibalicus* sp. nov. The asterisk (*) indicates the significance of the observed correlation.
(TIF)

S1 Table Detailed information on the specimens used in the phylogenetic analyses with locality data and GenBank accession numbers. Voucher codes of specimens available in collections refer to the following collections: IBE[X]: field series of S. Carranza housed at the Institute of Evolutionary Biology (CSIC-UPF); ONHM[X]: Oman Natural History Museum; NHMC[X]: Natural History Museum of Crete. Greece; NHMUK[X]: Natural History Museum of United Kingdom. The holotype (*) and paratypes are underlined.
(DOCX)

S2 Table List of primers used in the amplification and sequencing of gene fragments with the corresponding source and PCR conditions. Primer orientation (OR); F = forward, R = reverse.
(DOCX)

S3 Table Sex, metric and meristic variables measured and Morphobank accession numbers for the pictures of all examined specimens of *P*. *ruusaljibalicus* sp. nov. (1) and *P*. *orlovi* (2). The holotype (*) and paratypes are underlined. Museum acronyms as in S1 Table. SVL, Snout-vent length; AGL, Axilla-groin length; HL, Head length; HW, Head width; HH, Head height; END, Eye to nostril distance; IOD, Interorbital distance; OD, Orbital diameter; EED, Ear to eye distance; BL, Brachium length; AL, Antebrachium length; IVM, Digit IV of the manus; ThL, Thigh length; CL, Crus length; IVP, Digit IV of the pes; TubW, Number of rows of enlarged tubercles on the dorsum; TubA, Cloacal tubercles (distribution of cloacal tubercles on hemipenial bulges given in brackets); SL, Supralabial scales; IL, Infralabial scales; Fan4A, Terminal lamellae under the 4th finger; LF4, Subdigital scales on the 4th finger; Fan4P, Terminal lamellae under the 4th toe; LT4, Subdigital scales on the 4th toe.
(DOCX)
