# Supplementary figures and images for: Cryptic diversity in Ptyodactylus (Reptilia: Gekkonidae) from the northern Hajar Mountains of Oman and the United Arab Emirates uncovered by an integrative taxonomic approach
Source: PLoS One. 2017 Aug 2;12(8):e0180397. doi: 10.1371/journal.pone.0180397 (PMC5540286; doi:10.1371/journal.pone.0180397)

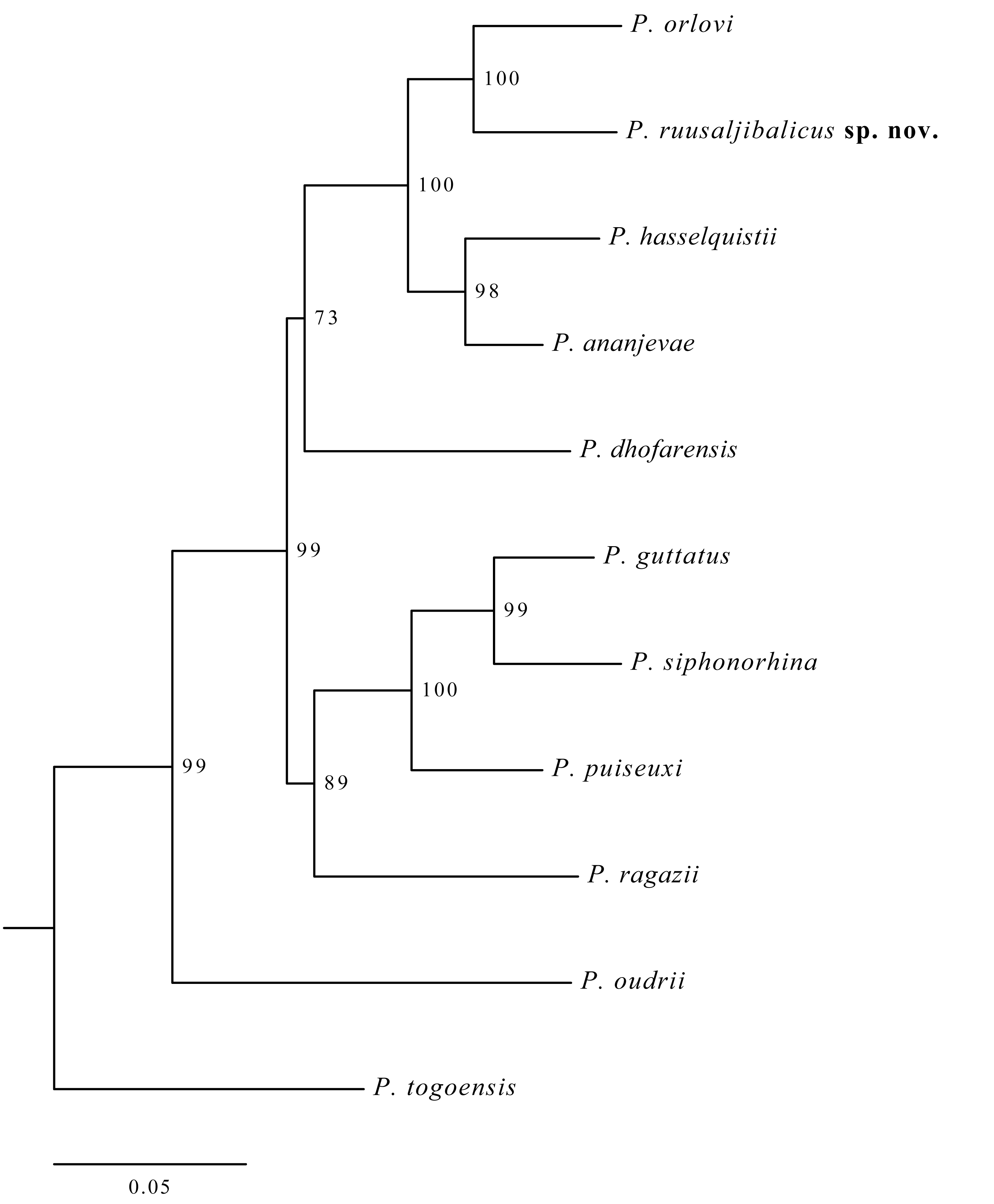

Supplement: S1 Fig — The phylogeny is based on the concatenated sequences of two mitochondrial (12S and cytb) and four nuclear (c-mos, MC1R, ACM4 and RAG2) gene fragments. Bootstrap values ≥70% of the ML analysis are shown next to the nodes. (TIF) [file pone.0180397.s001.tif]

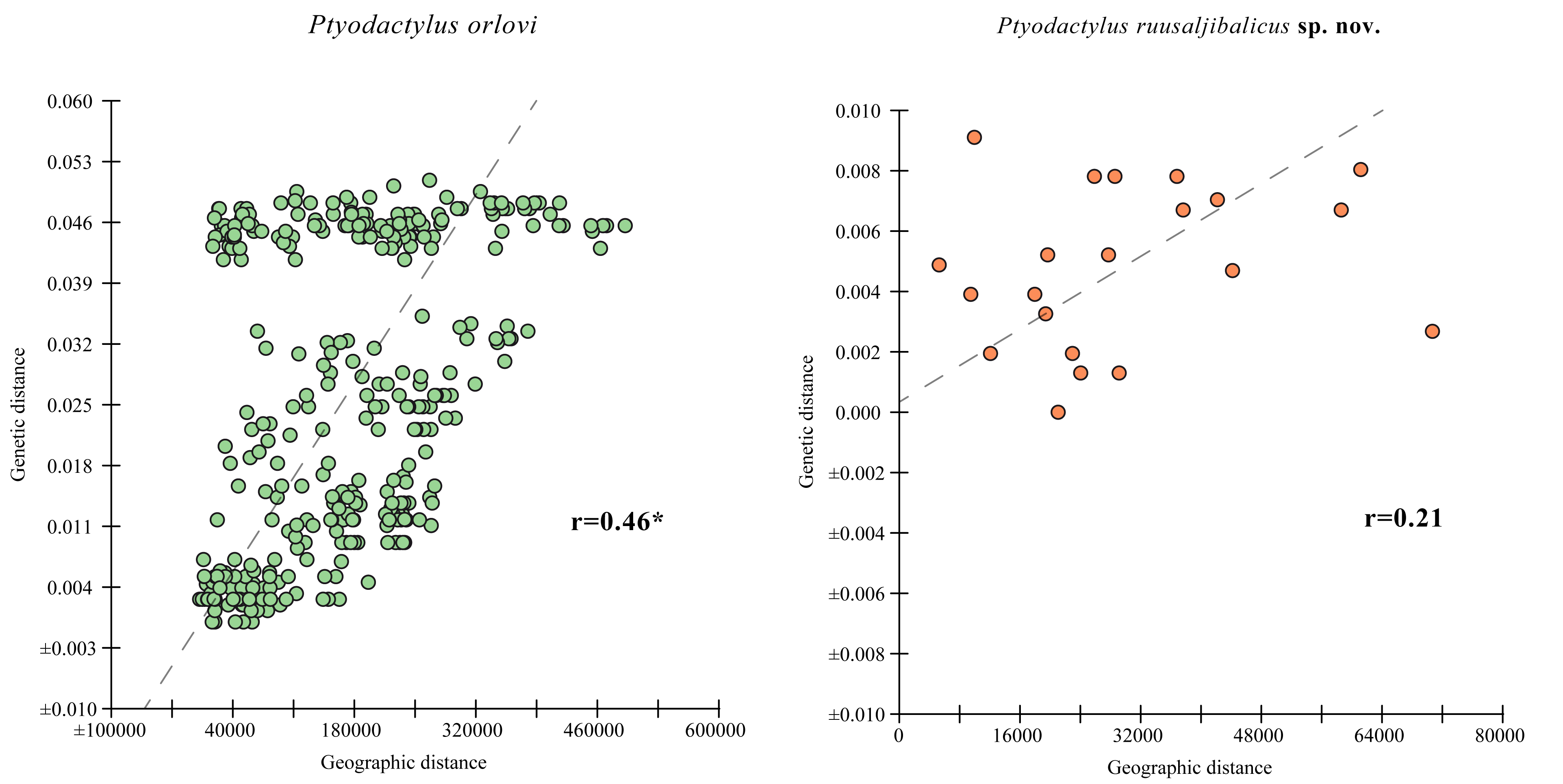

Supplement: S2 Fig — The asterisk (*) indicates the significance of the observed correlation. (TIF) [file pone.0180397.s002.tif]
