# Supplementary material for: Cryptic diversity in Ptyodactylus (Reptilia: Gekkonidae) from the northern Hajar Mountains of Oman and the United Arab Emirates uncovered by an integrative taxonomic approach
Source: PLoS One. 2017 Aug 2;12(8):e0180397. doi: 10.1371/journal.pone.0180397 (PMC5540286; doi:10.1371/journal.pone.0180397)
Supplement: S1 Table — Voucher codes of specimens available in collections refer to the following collections: IBE[X]: field series of S. Carranza housed at the Institute of Evolutionary Biology (CSIC-UPF); ONHM[X]: Oman Natural History Museum; NHMC[X]: Natural History Museum of Crete. Greece; NHMUK[X]: Natural History Museum of United Kingdom. The holotype (*) and paratypes are underlined. (DOCX) [file pone.0180397.s003.docx]

**S1 Table. Detailed information on the specimens used in the phylogenetic analyses with locality data and GenBank accession numbers.** Voucher codes of specimens available in collections refer to the following collections: IBE[X]: field series of S. Carranza housed at the Institute of Evolutionary Biology (CSIC-UPF); ONHM[X]: Oman Natural History Museum; NHMC[X]: Natural History Museum of Crete. Greece; NHMUK[X]: Natural History Museum of United Kingdom. The holotype (*) and paratypes are underlined.

| **Ingroup species** | **Specimen code** | **Voucher code** | **Country** | **Latitude** | **Longitude** | **Locality** | ***12S*** | ***cytb*** | ***c-mos*** | ***MC1R*** | ***ACM4*** | ***RAG2*** |
| --- | --- | --- | --- | --- | --- | --- | --- | --- | --- | --- | --- | --- |
| *P. ruusaljibalicus* **sp.nov.** | CN5959 | NHMUK2013.348 | Oman | 26.22711 | 56.21312 | 1 | MF084465 | MF084700 | MF084526 | MF084759 | MF084586 | MF084647 |
| *P. ruusaljibalicus* **sp.nov.** | CN892 | IBECN892 | Oman | 26.15057 | 56.16159 | 2 | MF084460 | MF084695 | MF084521 | MF084755 | MF084582 | MF084642 |
| *P. ruusaljibalicus* **sp.nov.** | UAE22 |  | Oman | 26.09917 | 56.32944 | 3 | KP858217 | KP867979 | KP868086 | KP868518 | KP868203 | KP868396 |
| *P. ruusaljibalicus* **sp.nov.** | CN3951 | NHMUK2013.347* | Oman | 26.04214 | 56.36966 | 4 | MF084456 | MF084691 | MF084517 | MF084751 | MF084578 | MF084638 |
| *P. ruusaljibalicus* **sp.nov.** | TW1011 |  | UAE | 25.97632 | 56.15225 | 5 | KP858460 | KP868059 | KP868174 | KP868520 | KP868282 | KP868397 |
| *P. ruusaljibalicus* **sp.nov.** | CN9029 | IBECN9029 | UAE | 25.97632 | 56.15225 | 5 | MF084461 | MF084696 | MF084522 | MF084756 | MF084583 | MF084643 |
| *. ruusaljibalicus* **sp.nov.** | CN8712 | IBECN8712 | UAE | 25.97632 | 56.15225 | 5 | MF084459 | MF084694 | MF084520 | MF084754 | MF084581 | MF084641 |
| *P. ruusaljibalicus* **sp.nov.** | CN8699 | IBECN8699 | UAE | 25.97632 | 56.15225 | 5 | MF084463 | MF084698 | MF084524 | MF084758 | MF084585 | MF084645 |
| *P. ruusaljibalicus* **sp.nov.** | CN9013 |  | Oman | 25.97805 | 56.20497 | 6 | MF084464 | MF084699 | MF084525 | - | - | MF084646 |
| *P. ruusaljibalicus* **sp.nov.** | CN8178 | ONHM3743 | Oman | 25.97805 | 56.20497 | 6 | MF084462 | MF084697 | MF084523 | MF084757 | MF084584 | MF084644 |
| *P. ruusaljibalicus* **sp.nov.** | CN8203 | IBECN8203 | Oman | 25.89705 | 56.23464 | 7 | MF084458 | MF084693 | MF084519 | MF084753 | MF084580 | MF084640 |
| *P. ruusaljibalicus* **sp.nov.** | CN8173 | IBECN8173 | Oman | 25.89705 | 56.23464 | 7 | MF084457 | MF084692 | MF084518 | MF084752 | MF084579 | MF084639 |
| *P. ruusaljibalicus* **sp.nov.** | TW1030 |  | UAE | 25.61392 | 56.02956 | 8 | MF084466 | KP868060 | KP868148 | KP868519 | KP868259 | KP868398 |
| *P. orlovi* | CN9030 | IBECN9030 | UAE | 25.45915 | 56.18369 | 9 | MF084426 | MF084667 | MF084486 | MF084721 | MF084547 | MF084607 |
| *P. orlovi* | CN9028 | IBECN9028 | UAE | 25.45915 | 56.18369 | 9 | MF084425 | MF084666 | MF084485 | MF084720 | MF084546 | MF084606 |
| *P. orlovi* | CN9016 | IBECN9016 | UAE | 25.45915 | 56.18369 | 9 | MF084432 | MF084673 | MF084492 | MF084727 | MF084553 | MF084613 |
| *P. orlovi* | CN4051 |  | UAE | 25.45915 | 56.18369 | 9 | MF084422 | MF084664 | MF084482 | MF084717 | MF084543 | MF084603 |
| *P. orlovi* | UAE37 |  | UAE | 25.22556 | 56.31611 | 10 | KP858222 | KP867975 | KP868088 | KP868514 | KP868205 | KP868393 |
| *P. orlovi* | CN9021 | IBECN9021 | UAE | 25.18386 | 56.22811 | 11 | MF084429 | MF084670 | MF084489 | MF084724 | MF084550 | MF084610 |
| *P. orlovi* | CN8695 | IBECN8695 | UAE | 25.18386 | 56.22811 | 11 | MF084433 | MF084674 | MF084493 | MF084728 | MF084554 | MF084614 |
| *P. orlovi* | CN7943 | IBECN7943 | UAE | 25.18386 | 56.22811 | 11 | MF084431 | MF084672 | MF084491 | MF084726 | MF084552 | MF084612 |
| *P. orlovi* | TW1010 |  | UAE | 25.1499 | 56.1411 | 12 | KP858459 | MF084684 | MF084510 | - | MF084571 | MF084631 |
| *P. orlovi* | TW1014 |  | UAE | 25.12228 | 56.20831 | 13 | KP858408 | MF084688 | MF084514 | MF084748 | MF084575 | MF084635 |
| *P. orlovi* | TW1013 |  | UAE | 25.12228 | 56.20831 | 13 | KP858407 | MF084687 | MF084513 | MF084747 | MF084574 | MF084634 |
| *P. orlovi* | TW1019 |  | UAE | 25.01703 | 56.06094 | 14 | KP858410 | MF084686 | MF084512 | MF084746 | MF084573 | MF084633 |
| *P. orlovi* | TW1015 |  | UAE | 25.00768 | 56.21518 | 15 | KP858409 | MF084689 | MF084515 | MF084749 | MF084576 | MF084636 |
| *P. orlovi* | CN4068 |  | UAE | 25.00768 | 56.21518 | 15 | MF084430 | MF084671 | MF084490 | MF084725 | MF084551 | MF084611 |
| *P. orlovi* | TW1012 |  | UAE | 24.99136 | 56.21969 | 16 | KP858406 | MF084685 | MF084511 | MF084745 | MF084572 | MF084632 |
| *P. orlovi* | OM7 |  | Oman | 24.75167 | 56.1789 | 17 | KP858469 | KP867976 | KP868178 | KP868515 | KP868286 | KP868394 |
| *P. orlovi* | CN239 | IBECN239 | Oman | 24.63515 | 56.29345 | 18 | MF084419 | MF084661 | MF084479 | MF084714 | MF084540 | MF084600 |
| *P. orlovi* | CN708 | IBECN708 | Oman | 24.62052 | 56.33947 | 19 | MF084424 | MF084665 | MF084484 | MF084719 | MF084545 | MF084605 |
| *P. orlovi* | CN668 | IBECN668 | Oman | 24.45284 | 56.29739 | 20 | - | MF084690 | MF084516 | MF084750 | MF084577 | MF084637 |
| *P. orlovi* | CN652 | IBECN652 | Oman | 24.44905 | 56.31647 | 21 | MF084423 | - | MF084483 | MF084718 | MF084544 | MF084604 |
| *P. orlovi* | CN2960 | IBECN2960 | Oman | 24.44905 | 56.31647 | 21 | MF084420 | MF084662 | MF084480 | MF084715 | MF084541 | MF084601 |
| *P. orlovi* | CN3879 | IBECN3879 | Oman | 23.71229 | 56.44176 | 22 | MF084428 | MF084669 | MF084488 | MF084723 | MF084549 | MF084609 |
| *P. orlovi* | CN3780 | IBECN3780 | Oman | 23.71229 | 56.44176 | 22 | MF084427 | MF084668 | MF084487 | MF084722 | MF084548 | MF084608 |
| *P. orlovi* | CN3911 | IBECN3911 | Oman | 23.79837 | 56.98738 | 23 | MF084421 | MF084663 | MF084481 | MF084716 | MF084542 | MF084602 |
| *P. orlovi* | CN3411 | IBECN3411 | Oman | 23.51514 | 56.41022 | 24 | MF084413 | MF084655 | MF084473 | MF084708 | MF084534 | MF084594 |
| *P. orlovi* | CN722 | IBECN722 | Oman | 23.14979 | 56.89423 | 25 | MF084415 | MF084657 | MF084475 | MF084710 | MF084536 | MF084596 |
| *P. orlovi* | CN666 |  | Oman | 23.14979 | 56.89423 | 25 | MF084417 | MF084659 | MF084477 | MF084712 | MF084538 | MF084598 |
| *P. orlovi* | CN236 | IBECN236 | Oman | 23.17412 | 57.03432 | 26 | MF084412 | MF084654 | MF084472 | MF084707 | MF084533 | MF084593 |
| *P. orlovi* | CN3872 |  | Oman | 23.19823 | 57.1381 | 27 | MF084414 | MF084656 | MF084474 | MF084709 | MF084535 | MF084595 |
| *P. orlovi* | CN3954 | IBECN3954 | Oman | 23.193 | 57.19918 | 28 | MF084418 | MF084660 | MF084478 | MF084713 | MF084539 | MF084599 |
| *P. orlovi* | CN3950 |  | Oman | 23.193 | 57.19918 | 28 | MF084416 | MF084658 | MF084476 | MF084711 | MF084537 | MF084597 |
| *P. orlovi* | CN926 | IBECN926 | Oman | 23.38342 | 57.30623 | 29 | MF084446 | MF084681 | MF084506 | MF084741 | MF084567 | MF084627 |
| *P. orlovi* | OM04/2010-12 |  | Oman | 23.33194 | 57.48916 | 30 | KP858441 | MF084682 | MF084508 | MF084743 | MF084569 | MF084629 |
| *P. orlovi* | OM04/2010-19 |  | Oman | 23.24361 | 57.4325 | 31 | KP858443 | MF084683 | MF084509 | MF084744 | MF084570 | MF084630 |
| *P. orlovi* | ZFMK70963 |  | Oman | 23.199 | 57.383 | 32 | KP858162 | KP868035 | KP868066 | KP868462 | KP868187 | KP868359 |
| *P. orlovi* | CN3949 | IBECN3949 | Oman | 23.06904 | 57.47324 | 33 | MF084409 | MF084651 | MF084469 | MF084704 | MF084530 | MF084590 |
| *P. orlovi* | CN3918 | IBECN3918 | Oman | 23.06904 | 57.47324 | 33 | MF084408 | MF084650 | MF084468 | MF084703 | MF084529 | MF084589 |
| *P. orlovi* | CN830 |  | Oman | 22.93289 | 57.66921 | 34 | MF084411 | MF084653 | MF084471 | MF084706 | MF084532 | MF084592 |
| *P. orlovi* | AO60 |  | Oman | 22.99722 | 57.70333 | 35 | KP858240 | KP867973 | KP868097 | KP868512 | KP868212 | KP868391 |
| *P. orlovi* | CN3309 | IBECN3309 | Oman | 23.44559 | 57.88009 | 36 | MF084434 | - | MF084494 | MF084729 | MF084555 | MF084615 |
| *P. orlovi* | CN149 |  | Oman | 23.44559 | 57.88009 | 36 | MF084407 | MF084649 | MF084467 | MF084702 | MF084528 | MF084588 |
| *P. orlovi* | CN8163 |  | Oman | 22.62568 | 58.30032 | 37 | MF084447 | - | MF084507 | MF084742 | MF084568 | MF084628 |
| *P. orlovi* | CN8140 |  | Oman | 22.62568 | 58.30032 | 37 | MF084444 | MF084679 | MF084504 | MF084739 | MF084565 | MF084625 |
| *P. orlovi* | CN7596 | IBECN7596 | Oman | 22.62568 | 58.30032 | 37 | MF084443 | MF084678 | MF084503 | MF084738 | MF084564 | MF084624 |
| *P. orlovi* | CN855 | IBECN855 | Oman | 23.0861 | 58.87609 | 38 | MF084445 | MF084680 | MF084505 | MF084740 | MF084566 | MF084626 |
| *P. orlovi* | CN779 | IBECN779 | Oman | 23.0861 | 58.87609 | 38 | MF084410 | MF084652 | MF084470 | MF084705 | MF084531 | MF084591 |
| *P. orlovi* | CN767 | IBECN767 | Oman | 23.0861 | 58.87609 | 38 | MF084439 | MF084675 | MF084499 | MF084734 | MF084560 | MF084620 |
| *P. orlovi* | CN771 | IBECN771 | Oman | 23.06756 | 59.01256 | 39 | MF084435 | - | MF084495 | MF084730 | MF084556 | MF084616 |
| *P. orlovi* | CN680 | IBECN680 | Oman | 22.95383 | 59.16826 | 40 | MF084438 | - | MF084498 | MF084733 | MF084559 | MF084619 |
| *P. orlovi* | CN8121 | IBECN8121 | Oman | 22.49632 | 58.68551 | 41 | MF084442 | MF084677 | MF084502 | MF084737 | MF084563 | MF084623 |
| *P. orlovi* | UAE41 |  | Oman | 22.62028 | 59.09306 | 42 | KP858219 | KP867974 | KP868087 | KP868513 | KP868204 | KP868392 |
| *P. orlovi* | CN858 |  | Oman | 22.5391 | 59.36788 | 43 | MF084436 | - | MF084496 | MF084731 | MF084557 | MF084617 |
| *P. orlovi* | CN3448 | IBECN3448 | Oman | 22.47915 | 59.28339 | 44 | MF084437 | - | MF084497 | MF084732 | MF084558 | MF084618 |
| *P. orlovi* | CN4066 | IBECN4066 | Oman | 22.40808 | 59.22364 | 45 | MF084440 | - | MF084500 | MF084735 | MF084561 | MF084621 |
| *P. orlovi* | CN4079 | IBECN4079 | Oman | 22.16919 | 59.41356 | 46 | MF084441 | MF084676 | MF084501 | MF084736 | MF084562 | MF084622 |
| *P. ananjevae* | Jor44 |  | Jordan | 29.633 | 35.5 |  | KP858465 | KP868018 | KP868176 | KP868439 | KP868284 | KP868336 |
| *P. dhofarensis* | OM04/2010-56 |  | Oman | 16.8794 | 53.7744 |  | KP858446 | KP868004 | KP868164 | KP868422 | KP868272 | KP868318 |
| *P. guttatus* | BEV.T394 |  | Egypt | 29.2608 | 32.9374 |  | KP858200 | KP868000 | KP868083 | KP868418 | KP868201 | KP868314 |
| *P. hasselquistii* | S4198 |  | Egypt | 30.011 | 31.248 |  | KP858449 | KP868024 | KP868166 | KP868451 | KP868274 | KP868348 |
| *P. oudrii* | BEV.T702 |  | Algeria | 33.75861 | 1.215 |  | KP858152 | KP867983 | KP868065 | KP868403 | KP868186 | KP868295 |
| *P. puiseuxi* | NHMC80.3.49.15 | NHMC80.3.49.15 | Jordan | 32.6108 | 35.7074 |  | KP858247 | KP867993 | KP868099 | KP868413 | KP868213 | KP868306 |
| *P. ragazzii* | TMHC179 |  | Ethiopia | 12.96081 | 40.97342 |  | KP858417 | KP867990 | KP868155 | KP868411 | KP868266 | KP868303 |
| *P. siphonorhina* | SPM002952-48 |  | Egypt | 29.95713 | 31.33666 |  | KP858237 | KP867954 | KP868095 | KP868493 | KP868210 | KP868372 |
| *P. togoensis* | BEV8870 |  | Mali | 19.17 | 7.78 |  | KP858196 | - | KP868081 | KP868400 | KP868199 | KP868291 |
| **Outgroup species** |  |  |  |  |  |  |  |  |  |  |  |  |
| *A. gallagheri* | CN4310 | IBECN4310 | Oman |  |  |  | KX550526 | KX550534 | KX550621 | KX550621 | KX550707 | - |
