# Supplementary material for: Cryptic diversity in Ptyodactylus (Reptilia: Gekkonidae) from the northern Hajar Mountains of Oman and the United Arab Emirates uncovered by an integrative taxonomic approach
Source: PLoS One. 2017 Aug 2;12(8):e0180397. doi: 10.1371/journal.pone.0180397 (PMC5540286; doi:10.1371/journal.pone.0180397)
Supplement: S2 Table — Primer orientation (OR); F = forward, R = reverse. (DOCX) [file pone.0180397.s004.docx]

| **Locus** | **Primer name** | **OR** | **Primer sequence (5’- 3’)** | **Primer source** | **PCR conditions** |
| --- | --- | --- | --- | --- | --- |
| *12S* | 12SaGekko | F | CAAACTAGGATTAGATACCCTACTATGC | Metallinou et al. (2015) | 94º(5’);94º(45”), 52º(45”), 72º(80”) x 35;72(5’) |
|  | 12SbGekko | R | GAGGGTGACGGGCGGTGTGTAC | Metallinou et al. (2015) |  |
| *cytb* | Salvi1 | F | TCCAACTACAAAAACCTAATGACCC | Metallinou et al. (2015) | 94º(5’);94º(30”), 48º(45”), 72º(60”) x 35;72º(5’) |
|  | cytb2 | R | CCCTCAGAATGATATTTGTCCTCA | Palumbi et al. (1991) |  |
| *c-mos* | CMOS-FUF | F | TTTGGTTCKGTCTACAAGGCTAC | Gamble et al. (2008) | 94º (5’);94º(45”), 55º (45”), 72º (70”) x 40;72º (10’) |
|  | CMOS-FUR | R | AGGGAACATCCAAAGTCTCCAAT | Gamble et al. (2008) |  |
| *MC1R* | MC1R-F | F | GGCNGCCATYGTCAAGAACCGGAACC | Pinho et al. (2009) | 94º(5’);94º(30”), 52º(45”), 72º (90”) x 40;72º(5’) |
|  | MC1R-R | R | CTCCGRAAGGCRTAAATGATGGGGTCCAC | Pinho et al. (2009) |  |
| *ACM4* | ACM4-TgF | F | CAAGCCTGAGCAARAAGG | Gamble et al. (2008) | 94º (5’);94º(45”), 55º (45”), 72º (80”) x 40;72º (5’) |
|  | ACM4-TgR | R | ACYTGACTCCTGGCAATGCT | Gamble et al. (2008) |  |
| *RAG2* | RAG2-PY1F | F | CCCTGAGTTTGGATGCTGTACTT | Gamble et al. (2008) | 94º (5’);94º(45”), 55º (45”), 72º (80”) x 40;72º (5’) |
|  | RAG2-PY1R | R | AACTGCCTRTTGTCCCCTGGTAT | Gamble et al. (2008) |  |

**S2 Table. List of primers used in the amplification and sequencing of gene fragments, with the corresponding source and PCR conditions.** Primer orientation (OR); F=forward, R=reverse.

**References to Table S2.**

Gamble T, Bauer AM, Greenbaum E, Jackman TR. Evidence for Gondwanan vicariance in an ancient clade of gecko lizards. J Biogeogr. 2008; 35: 88–104.

Metallinou M, Červenka J, Crochet P-A, Kratochvíl L, Wilms T, Geniez P, et al. Species on the rocks: Systematics and biogeography of the rock-dwelling *Ptyodactylus* geckos (Squamata: Phyllodactylidae) in North Africa and Arabia. Mol Phylogenet Evol. 2015; 85: 208–220.

Palumbi, S.R., Martin, A.P., Romano, S., McMillan, W.O., Stice, L., Grabowski, G. The Simple Fool’s Guide to PCR. Department of Zoology Special Publication, University of Hawaii, Honolulu, HI. 1991.

Pinho C, Rocha S, Carvalho BM, Lopes S, Mourao S et al. New primers for the amplification and sequencing of nuclear loci in a taxonomically wide set of reptiles and amphibians. Conserv Genet Resour. 2009; 2: 181-185.
