# Supplementary material for: Cryptic diversity in Ptyodactylus (Reptilia: Gekkonidae) from the northern Hajar Mountains of Oman and the United Arab Emirates uncovered by an integrative taxonomic approach
Source: PLoS One. 2017 Aug 2;12(8):e0180397. doi: 10.1371/journal.pone.0180397 (PMC5540286; doi:10.1371/journal.pone.0180397)
Supplement: S3 Table — The holotype (*) and paratypes are underlined. Museum acronyms as in S1 Table. SVL, Snout-vent length; AGL, Axilla-groin length; HL, Head length; HW, Head width; HH, Head height; END, Eye to nostril distance; IOD, Interorbital distance; OD, Orbital diameter; EED, Ear to eye distance; BL, Brachium length; AL, Antebrachium length; IVM, Digit IV of the manus; ThL, Thigh length; CL, Crus length; IVP, Digit IV of the pes; TubW, Number of rows of enlarged tubercles on the dorsum; TubA, Cloacal tubercles (distribution of cloacal tubercles on hemipenial bulges given in brackets); SL, Supralabial scales; IL, Infralabial scales; Fan4A, Terminal lamellae under the 4th finger; LF4, Subdigital scales on the 4th finger; Fan4P, Terminal lamellae under the 4th toe; LT4, Subdigital scales on the 4th toe. (DOCX) [file pone.0180397.s005.docx]

**S3 Table.** **Sex, metric and meristic variables measured and Morphobank accession numbers for the pictures of all examined specimens of *P. ruusaljibalicus* sp. nov. (1) and *P. orlovi* (2).** The holotype (*) and paratypes are underlined. Museum acronyms as in S1 Table. SVL, Snout-vent length; AGL, Axilla-groin length; HL, Head length; HW, Head width; HH, Head height; END, Eye to nostril distance; IOD, Interorbital distance; OD, Orbital diameter; EED, Ear to eye distance; BL, Brachium length; AL, Antebrachium length; IVM, Digit IV of the manus; ThL, Thigh length; CL, Crus length; IVP, Digit IV of the pes; TubW, Number of rows of enlarged tubercles on the dorsum; TubA, Cloacal tubercles (distribution of cloacal tubercles on hemipenial bulges given in brackets); SL, Supralabial scales; IL, Infralabial scales; Fan4A, Terminal lamellae under the 4^th^ finger; LF4, Subdigital scales on the 4^th^ finger; Fan4P, Terminal lamellae under the 4^th^ toe; LT4, Subdigital scales on the 4^th^ toe.

| **Species** | **Voucher code** | **Sex** | **SVL** | **AGL** | **HL** | **HW** | **HH** | **END** | **IOD** | **OD** | **EED** | **BL** | **AL** | **IVM** | **ThL** | **CL** | **IVP** | **TubW** | **TubA** | **SL** | **IL** | **Fan4A** | **LF4** | **Fan4P** | **LT4** | **MorphoBank** |
| --- | --- | --- | --- | --- | --- | --- | --- | --- | --- | --- | --- | --- | --- | --- | --- | --- | --- | --- | --- | --- | --- | --- | --- | --- | --- | --- |
| 1 | NHMUK2013.347* | M | 80.2 | 33.76 | 21.57 | 14.83 | 8.7 | 7.49 | 7.42 | 5.64 | 5.68 | 14.16 | 16.92 | 6.69 | 20.84 | 19.17 | 7.67 | 11 | 4 (2/2) | 12 | 12 | 20 | 11 | 20 | 11 | M339669–M339684 |
| 1 | NHMUK2013.348 | M | 86.46 | 35.96 | 23.59 | 15.32 | 9.65 | 8.49 | 8.87 | 6.01 | 6.37 | 13.94 | 16.85 | 6.42 | 20.38 | 20.93 | 8.63 | 11 | 4 (2/2) | 13 | 12 | 20 | 11 | 22 | 10 | M339685–M339691 |
| 1 | IBECN892 | M | 90.01 | 39.41 | 24.54 | 15.86 | 10.21 | 8.84 | 7.66 | 5.63 | 6.7 | 12.8 | 17.11 | 7.71 | 20.62 | 21.18 | 10.34 | 11 | 4 (2/2) | 14 | 12 | 20 | 10 | 22 | 10 | M339662–M339668 |
| 1 | ONHM3743 | F | 85.92 | 35.27 | 23.14 | 16.36 | 9.65 | 8.32 | 8.14 | 5.45 | 7.22 | 13.76 | 16.64 | 7.68 | 17.92 | 19.84 | 9.84 | 11 | 4 (2/2) | 13 | 13 | 20 | 9 | 22 | 10 | M339698–M339717 |
| 1 | IBECN8173 | J | - | - | - | - | - | - | - | - | - | - | - | - | - | - | - | 11 | 4 (2/2) | 14 | 13 | 20 | 11 | 22 | 11 | M339692–M339697 |
| 1 | IBECN8203 | J | - | - | - | - | - | - | - | - | - | - | - | - | - | - | - | 9 | 4 (2/2) | 13 | 13 | 20 | 10 | 20 | 10 | M339718–M339723 |
| 1 | IBECN8699 | J | - | - | - | - | - | - | - | - | - | - | - | - | - | - | - | 9 | 4 (2/2) | 13 | 13 | 20 | 9 | 20 | 11 | M339724–M339728 |
| 1 | IBECN8712 | J | - | - | - | - | - | - | - | - | - | - | - | - | - | - | - | 10 | 4 (2/2) | 14 | 13 | 18 | 9 | 20 | 10 | M339729–M339733 |
| 1 | IBECN9029 | J | - | - | - | - | - | - | - | - | - | - | - | - | - | - | - | 10 | 4 (2/2) | 13 | 13 | 22 | 10 | 20 | 11 | M339734–M339739 |
| 2 | IBECN3411 | M | 81.92 | 36.39 | 21.61 | 15.53 | 9.78 | 7.36 | 6.55 | 5.47 | 6.65 | 14.14 | 16.01 | 7.37 | 20.84 | 19.53 | 8.92 | 13 | 4 (2/2) | 14 | 14 | 20 | 9 | 22 | 11 | M339825–M339830 |
| 2 | IBECN3448 | M | 80.3 | 33.4 | 22.19 | 15.33 | 9.35 | 7.1 | 6.67 | 5.62 | 6.63 | 13.13 | 16.15 | 8 | 21.12 | 18.94 | 8.59 | 12 | 4 (2/2) | 15 | 13 | 20 | 9 | 18 | 9 | M339831–M339833 |
| 2 | IBECN3780 | M | 81.73 | 32.25 | 23 | 15.9 | 9.75 | 7.53 | 7.13 | 5.45 | 6.43 | 13.83 | 15.62 | 6.77 | 19.99 | 19.65 | 7.99 | 12 | 1 (0/1) | 14 | 12 | 20 | 9 | 20 | 9 | M339834–M339839 |
| 2 | IBECN3911 | M | 73.66 | 32.92 | 20.53 | 14.1 | 8.52 | 6.79 | 5.9 | 5.46 | 5.6 | 11.96 | 13.58 | 7.36 | 18.76 | 17.66 | 7.86 | 13 | 3 (0/3) | 13 | 12 | 20 | 10 | 22 | 10 | M339846–M339851 |
| 2 | IBECN3949 | M | 80.09 | 36.72 | 22.38 | 15.7 | 9.16 | 7.35 | 6.54 | 5.68 | 6.62 | 12.88 | 15.15 | 7.09 | 20.36 | 19.63 | 8.07 | 11 | 4 (2/2) | 15 | 13 | 22 | 9 | 22 | 11 | M339858–M339863 |
| 2 | IBECN3954 | M | 89.46 | 38.53 | 23.41 | 15.99 | 9.63 | 8.4 | 6.57 | 5.89 | 7.18 | 12.43 | 16.79 | 6.89 | 20.82 | 21.06 | 8.13 | 13 | 6 (3/3) | 14 | 12 | 18 | 9 | 20 | 10 | M339864–M339869 |
| 2 | IBECN4066 | M | 74.7 | 32.09 | 22.11 | 14.22 | 9.31 | 7.08 | 5.99 | 5.2 | 6.15 | 11.81 | 15.37 | 6.29 | 20.03 | 17.08 | 7.93 | 12 | 6 (3/3) | 14 | 12 | 18 | 9 | 20 | 10 | M339870–M339873 |
| 2 | IBECN4079 | M | 87.23 | 37.27 | 23.45 | 16.17 | 10.59 | 8.25 | 7.18 | 6.33 | 7.32 | 13.62 | 16.59 | 7.44 | 23.99 | 20.5 | 8.88 | 12 | 3 (2/1) | 14 | 12 | 20 | 10 | 20 | 10 | M339874–M339879 |
| 2 | IBECN708 | M | 81.38 | 31.91 | 23.09 | 14.22 | 9.16 | 7.66 | 5.9 | 5.81 | 6.29 | 12.97 | 15.32 | 6.75 | 21.39 | 18.82 | 7.84 | 14 | 4 (2/2) | 14 | 13 | 20 | 10 | 22 | 10 | M339770–M339775 |
| 2 | IBECN771 | M | 81.09 | 33.81 | 22.47 | 14.94 | 9.91 | 7.84 | 6.66 | 5.75 | 6.4 | 13.44 | 16.38 | 8.05 | 22.59 | 21.26 | 8.52 | 12 | 4 (2/2) | 15 | 13 | 20 | 9 | 20 | 11 | M339788–M339793 |
| 2 | IBECN779 | M | 77.32 | 33.8 | 21.9 | 14.6 | 9.36 | 7.1 | 6.13 | 5.28 | 6.27 | 12.11 | 15.11 | 6.2 | 20.05 | 18.68 | 8.08 | 13 | 3 (2/1) | 13 | 13 | 20 | 9 | 22 | 10 | M339794–M339799 |
| 2 | IBECN855 | M | 89.25 | 33.22 | 24.72 | 16.91 | 10.7 | 8.78 | 8.51 | 6.13 | 7.29 | 13.1 | 17.03 | 7.96 | 20.13 | 20.53 | 9.03 | 13 | 3 (2/1) | 14 | 14 | 20 | 9 | 22 | 11 | M339800–M339805 |
| 2 | IBECN8695 | M | 83.47 | 34.07 | 23 | 15.23 | 9.41 | 7.78 | 6.87 | 6.09 | 7.39 | 13.82 | 15.97 | 7.54 | 21.47 | 19.65 | 8.22 | 12 | 5 (3/2) | 15 | 13 | 20 | 10 | 20 | 10 | M339898–M339904 |
| 2 | IBECN9021 | M | 86.08 | 35.55 | 24.39 | 15.89 | 9.72 | 7.85 | 6.5 | 5.72 | 7.48 | 13.33 | 15.86 | 6.48 | 21.82 | 19.88 | 8.67 | 14 | 5 (3/2) | 14 | 14 | 20 | 9 | 22 | 10 | M339911–M339918 |
| 2 | IBECN9028 | M | 84.95 | 35.45 | 23.9 | 15.36 | 9.8 | 8.07 | 6.84 | 5.86 | 7.2 | 13.62 | 16.78 | 7.63 | 21.82 | 19.12 | 9.39 | 14 | 4 (2/2) | 15 | 13 | 22 | 10 | 22 | 10 | M339919–M339925 |
| 2 | IBECN9030 | M | 81.81 | 34.48 | 22.09 | 14.75 | 8.81 | 8.15 | 6.66 | 5.94 | 6.64 | 13.89 | 14.98 | 6.95 | 19.81 | 18.69 | 8.09 | 13 | 2 (0/2) | 14 | 13 | 20 | 10 | 22 | 11 | M339926–M339931 |
| 2 | IBECN236 | F | 83.67 | 37.17 | 22.33 | 15.24 | 8.3 | 7.06 | 5.88 | 6.01 | 5.94 | 14.09 | 15.66 | 7.38 | 21.93 | 19.14 | 8.74 | 12 | 4 (2/2) | 14 | 15 | 20 | 10 | 22 | 11 | M339740–M339745 |
| 2 | IBECN239 | F | 73.64 | 32.39 | 20.51 | 13.44 | 8.75 | 6.81 | 5.65 | 5.3 | 5.6 | 11.45 | 14.51 | 5.69 | 17.91 | 16.49 | 7.23 | 13 | 1 (0/1) | 14 | 14 | 18 | 9 | 18 | 11 | M339747–M339752 |
| 2 | IBECN2960 | F | 79.4 | 36.45 | 21.38 | 14.66 | 9.41 | 7.35 | 6.11 | 5.56 | 6.28 | 13.27 | 15.77 | 6.58 | 21.69 | 18.07 | 7.49 | 13 | 3 (2/1) | 14 | 13 | 20 | 10 | 22 | 11 | M339814–M339819 |
| 2 | IBECN3309 | F | 78.39 | 34.53 | 21.12 | 14.69 | 9.68 | 7.74 | 6.78 | 5.72 | 6.15 | 11.4 | 15.31 | 6.85 | 19.09 | 18.81 | 7.35 | 11 | 2 (0/2) | 14 | 13 | 20 | 9 | 20 | 10 | M339820–M339824 |
| 2 | IBECN3879 | F | 67.83 | 29.31 | 19.1 | 13.44 | 7.79 | 6.17 | 6.04 | 4.8 | 5.47 | 12.46 | 13.71 | 6.18 | 19.32 | 16.06 | 7.62 | 12 | 5 (3/2) | 14 | 13 | 20 | 10 | 22 | 11 | M339840–M339845 |
| 2 | IBECN652 | F | 75.34 | 32.82 | 20.66 | 13.61 | 8.5 | 7.39 | 6.21 | 5.06 | 5.87 | 12.75 | 13.73 | 6.25 | 18.75 | 17.08 | 6.97 | 12 | 4 (2/2) | 15 | 13 | 18 | 10 | 20 | 11 | M339753–M339758 |
| 2 | IBECN668 | F | 83.28 | 34.93 | 22.67 | 14.28 | 9.49 | 7.53 | 6.13 | 5.49 | 6.02 | 13.19 | 15.43 | 6.66 | 20.66 | 20.45 | 7.42 | 11 | 4 (2/2) | 12 | 12 | 20 | 9 | 22 | 10 | M339759–M339764 |
| 2 | IBECN680 | F | 81.25 | 35.66 | 23.35 | 15.57 | 10.22 | 7.84 | 6.51 | 5.73 | 6.46 | 13.17 | 16.35 | 8.23 | 18.71 | 19.13 | 9.36 | 13 | 2 (0/2) | 14 | 14 | 20 | 9 | 22 | 11 | M339765–M339769 |
| 2 | IBECN722 | F | 84.17 | 37.96 | 22.76 | 14.69 | 9.35 | 7.6 | 6.39 | 5.75 | 5.91 | 13.39 | 15.57 | 6.39 | 21 | 18.96 | 7.54 | 13 | 1 (0/1) | 15 | 13 | 18 | 10 | 20 | 11 | M339776–M339781 |
| 2 | IBECN7596 | F | 80.66 | 35.28 | 21.8 | 14.52 | 9.11 | 7.47 | 6.61 | 5.92 | 5.66 | 12.21 | 16.18 | 6.39 | 16.74 | 18.26 | 7.23 | 14 | 4 (2/2) | 14 | 13 | 22 | 10 | 22 | 11 | M339880–M339884 |
| 2 | IBECN767 | F | 82.13 | 33.27 | 22.85 | 15.43 | 9.57 | 7.25 | 6.38 | 5.59 | 6.62 | 13.45 | 16.95 | 6.77 | 21.58 | 20.01 | 7.52 | 12 | 3 (2/1) | 13 | 12 | 20 | 10 | 20 | 10 | M339782–M339787 |
| 2 | IBECN7943 | F | 85.1 | 35.68 | 22.49 | 15.67 | 9.73 | 8.1 | 7.12 | 5.64 | 6.5 | 13.22 | 14.33 | 7.22 | 19.79 | 19.65 | 8.99 | 13 | 3 (2/1) | 14 | 13 | 20 | 10 | 20 | 12 | M339885–M339891 |
| 2 | IBECN926 | F | 70.32 | 30.37 | 19.44 | 13.63 | 8.21 | 7.17 | 6.97 | 5.52 | 5.58 | 12.13 | 14.06 | 6.49 | 19.18 | 16.64 | 8.24 | 12 | 5 (2/3) | 14 | 13 | 20 | 9 | 20 | 12 | M339806–M339813 |
| 2 | IBECN3918 | J | - | - | - | - | - | - | - | - | - | - | - | - | - | - | - | 12 | 4 (2/2) | 15 | 13 | 18 | 9 | 20 | 12 | M339852–M339857 |
| 2 | IBECN8121 | J | - | - | - | - | - | - | - | - | - | - | - | - | - | - | - | 12 | 2 (1/1) | 13 | 12 | 20 | 10 | 20 | 11 | M339892–M339897 |
| 2 | IBECN9016 | J | - | - | - | - | - | - | - | - | - | - | - | - | - | - | - | 13 | 6 (3/3) | 13 | 14 | 22 | 9 | 22 | 11 | M339905–M339910 |
